# SAM68 promotes tumorigenesis in lung adenocarcinoma by regulating metabolic conversion via PKM alternative splicing

**DOI:** 10.7150/thno.51360

**Published:** 2021-01-19

**Authors:** Song Zhu, Weiping Chen, Jizhong Wang, Ling Qi, Huilin Pan, Zhengfu Feng, Dongbo Tian

**Affiliations:** 1 Department of Radiotherapy, The Sixth Affiliated Hospital of Guangzhou Medical University, Qingyuan People's Hospital, Qingyuan 511518, P. R. China.; 2 Department of Respiratory, The Sixth Affiliated Hospital of Guangzhou Medical University, Qingyuan People's Hospital, Qingyuan 511518, P. R. China.; 3 Department of Cardiology, Vascular Center, Guangdong Cardiovascular Institute, Guangdong Provincial People's Hospital, Guangdong Academy of Medical Sciences, Guangdong, People's Republic of China.; 4 Department of Central Laboratory, The Sixth Affiliated Hospital of Guangzhou Medical University, Qingyuan People's Hospital, Qingyuan 511518, P. R. China.

**Keywords:** SAM68, alternative splicing, metabolism conversion, tumorigenesis, lung adenocarcinoma

## Abstract

**Background:** A metabolic “switch” from oxidative phosphorylation to glycolysis provides tumor cells with energy and biosynthetic substrates, thereby promoting tumorigenesis and malignant progression. However, the mechanisms controlling this metabolic switch in tumors is not entirely clear.

**Methods:** Clinical specimens were used to determine the effect of SAM68 on lung adenocarcinoma (LUAD) tumorigenesis and metastasis, and mouse models and molecular biology assays were performed to elucidate the function and underlying mechanisms *in vitro* and *in vivo*.

**Results:** *SAM68* mRNA levels were higher in LUAD tissue than in normal lung tissue, indicating that *SAM68* expression is upregulated in LUAD. Patients with LUAD with* SAM68*^high^ (n = 257) had a higher frequency of tumor recurrence (*p =* 0.025) and recurrence-free survival (*p =* 0.013) than did those with *SAM68*^low^ (n = 257). Patients with *SAM68*^high^ mRNA levels (n = 257) were at a higher risk for cancer-related death (*p =* 0.006), and had shorter overall survival (*p =* 0.044) than did those with *SAM68*^low^. SAM68 promotes tumorigenesis and metastasis of LUAD cells *in vitro* and *in vivo* by regulating the cancer metabolic switch. SAM68 drives cancer metabolism by mediating alternative splicing of pyruvate kinase (PKM) pre-mRNAs, and promoting the formation of PKM2. Mechanistically, SAM68 increased the binding of the splicing repressor hnRNP A1 to exon 9 of *PKM*, thereby enhancing PKM2 isoform formation and PKM2-dependent aerobic glycolysis and tumorigenesis.

**Conclusions:** SAM68 promotes LUAD cell tumorigenesis and cancer metabolic programming via binding of the 351-443 aa region of SAM68 to the RGG motif of hnRNP A1, driving hnRNP A1-dependent *PKM* splicing, contributing to increased oncogene PKM2 isoform formation and inhibition of PKM1 isoform formation. SAM68 is therefore a promising therapeutic target for the treatment of LUAD.

## Introduction

Lung adenocarcinoma (LUAD) is among the most common human malignancies, and its incidence worldwide continues to rapidly increase 1. Patients with early-stage LUAD have a good 5-year survival rate because they respond well to current treatments. However, the prognosis in patients with advanced disease remains poor owing to the high incidence of tumor recurrence and distant metastasis 2,3. There is therefore an urgent need to understand the underlying molecular mechanisms responsible for LUAD pathogenesis and to discover new prognostic biomarkers and therapeutic targets for LUAD.

KHDRBS1, or SRC-associated in mitosis, 68 kDa (SAM68), was originally identified as the first specific target of Src tyrosine kinase in mitosis 4-6. There is increasing evidence of the important role of SAM68 in alternative splicing. SAM68 modulates CD44 alternative splicing in response to mitoxantrone (MTX)-induced DNA damage 7. ERK phosphorylation of Sam68 enhances inclusion of the CD44 exon v5 sequence in mRNA 8. In addition, ERK1/2 signaling pathway phosphorylation of Sam68 regulates epithelial-mesenchymal transition (EMT) via modulation of SF2/ASF splicing 9. SAM68 binds Bcl-x mRNA and modulates its alternative splicing by interacting with hnRNPA1 10. Infection with dominant-negative SAM68 mutants with disrupted RNA-binding or impaired binding to the splicing repressor hnRNP A1 enhanced exon-7 inclusion in endogenous SMN2 and rescued SMN protein expression in fibroblasts of patients with SMA 11. Further, deletion of SAM68 in breast cancer cells led to cell-cycle arrest at the G1/S checkpoint, suggesting that SAM68 is a novel cell-cycle regulator 12. SAM68 is overexpressed in early-stage cervical cancer associated with lymph node metastasis 13. However, its role and molecular mechanisms in LUAD progression and metastasis remain unknown.

hnRNP A1 belongs to the hnRNP family, a large family of ubiquitously expressed heterogeneous nuclear ribonucleoproteins (hnRNPs) that regulate several aspects of nucleic acid metabolism, including pre-mRNA packing, alternative splicing, and transportation of poly(A) mRNA 14-16. hnRNP expression is altered in many types of cancers 17. hnRNP A1 regulates mitochondrial dynamics by post-transcriptional regulation of the oncogene *Drp1* 18, and is a potential marker for breast cancer progression 19; however, the molecular mechanism by which hnRNP A1 is upregulated in cancer remains unclear.

Cancer cells exhibit increased glucose consumption and lactate generation instead of mitochondrial oxidative phosphorylation independent of oxygen availability; this phenomenon is termed the Warburg effect or aerobic glycolysis 20. PKM1 and PKM2 are products of alternative splicing of the *PKM* gene (encoding the glycolytic enzyme pyruvate kinase), which is related to the metabolic phenotype of cancer cells 21. PKM2 expression has metabolic advantages in cancer cells and is a critical determinant of aerobic glycolysis, which is required for the rapid growth of cancer cells and tumorigenesis 22. The two PKM isoforms contain a mutually exclusive exon, reflecting the inclusion of either exon 9 (PKM1) or 10 (PKM2) 23. hnRNPA1, hnRNPA2, and PTBP1 bind to exon 9 (E9) to repress E9 in the PKM transcript, resulting in enhanced expression of the PKM2 isoform rather than the PKM1 isoform 23,24.

Here, we found that SAM68 was upregulated in LUAD tissues compared to adjacent non-tumor tissues. Patients with LUAD with high SAM68 levels had shorter survival times than did those with low SAM68 levels. SAM68 promotes metabolic reprogramming and tumorigenesis in cancer by stimulating hnRNP A1-dependent PKM splicing and subsequent PKM2 isoform formation by binding of the 351-443 aa region of SAM68 to the RGG motif of hnRNP A1. Our findings indicate that SAM68 promotes PKM2 isoform formation, aerobic glycolysis, and tumorigenesis in LUAD cells, and could show potential as a novel potential target for the treatment of LUAD.

## Results

### High SAM68 expression in patients with LUAD correlates with poor prognosis

**Figure 1:**
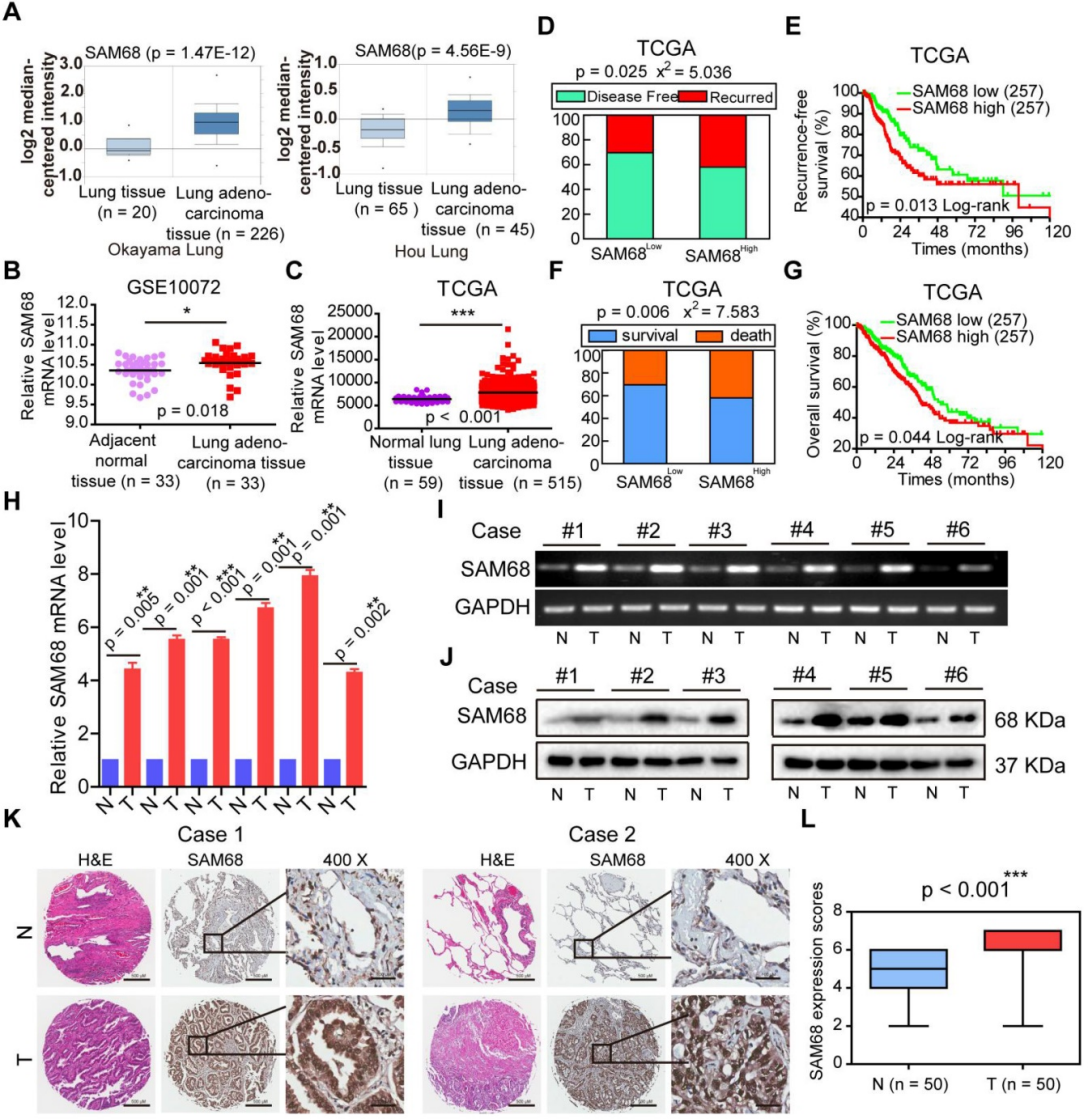
** SAM68 is up-regulated in Lung adenocarcinoma (LUAD) and correlates with a poor prognosis for LUAD patients.** (A~C) *SAM68* mRNA levels was up-regulated in LUAD compared to normal lung tissue based on the Oncomine, GEO and TCGA database. (D~G) From TCGA LUAD specimen cohorts, compared with the patients with low expression level of *SAM68*, the patients with high mRNA expression of* SAM68* had higher recurrence rates and death rates, shorter RFS, and OS. (H~J) The *SAM68*mRNA and protein levels were detected by qPCR, RT-PCR and Western blot in the LUAD tissues (T) and their corresponding adjacent non-tumoral tissues (N). (K) IHC analysis of the expression of SAM68 protein was represented in LUAD tissues and the corresponding adjacent normal lung tissues. (L) Differences in expression levels of SAM68 protein in LUAD tissues (n = 50) and adjacent normal lung tissues (n = 50). Two-tailed t-tests were used B, C, H and L. Pearson Chi-Square tests were used in D and F.Log rank tests were used in E and G.

To determine the role of *SAM68* in LUAD, we studied *SAM68* mRNA expression in normal lung and in LUAD tissues by using four microarray gene expression datasets from the Oncomine (https://www.oncomine.org), GEO (https://www.ncbi.nlm.nih.gov/geo/), and TCGA databases. *SAM68* mRNA levels were higher in LUAD tissues than in normal lung tissues (Figure 1A-C), indicating that *SAM68* expression is upregulated in LUAD.

To determine whether SAM68 influences the death and tumor recurrence rates, we analyzed the correlation between *SAM68* mRNA levels and the tumor recurrence and death rates in a publicly accessible microarray dataset from 515 patients with LUAD in the TCGA database cohort (provisional), downloaded from cBioPortal (http://www.cbioportal.org). *SAM68* mRNA level was set as a dichotomous variable, with *SAM68*^low^, defined as *SAM68* expression levels in the lower 50% of the cohort (n = 257), compared with *SAM68*^high^, which was defined as *SAM68* expression levels in the upper 50% of the cohort (n = 257). Chi-square and Kaplan-Meier survival analyses revealed that patients with LUAD with* SAM68*^high^ (n = 257) had a higher frequency of tumor recurrence and recurrence-free survival than did LUAD patients with *SAM68*^low^ (n = 257; Figure 1D-E). Moreover, patients with *SAM68*^high^ mRNA levels (n = 257) were at higher risk for cancer-related death, and had shorter overall survival than did *SAM68*^low^ patients (Figure 1F-G).

We also analyzed *SAM68* protein and mRNA levels in six sets of primary LUAD tissue samples and matched adjacent nontumor tissues (N). All LUAD tissues had elevated *SAM68* protein and mRNA levels compared to the corresponding N samples (Figure 1H-J). We also performed extensive microarray analysis of 50 LUAD and matched nontumor lung tissue (N) samples by using an immunohistochemical (IHC) assay (Figure 1K). The SAM68 protein level was higher in LUAD tissues than in N tissues (Figure 1L). Our findings indicate that SAM68 is significantly upregulated in LUAD cells, and patients with primary LUAD and *SAM68*^high^ exhibit a poor prognosis, suggesting that SAM68 could be a useful prognostic marker and potential therapeutic target in patients with LUAD.

### SAM68 promotes malignant phenotypes in LUAD cells *in vitro* and tumorigenesis and metastasis* in vivo*

**Figure 2:**
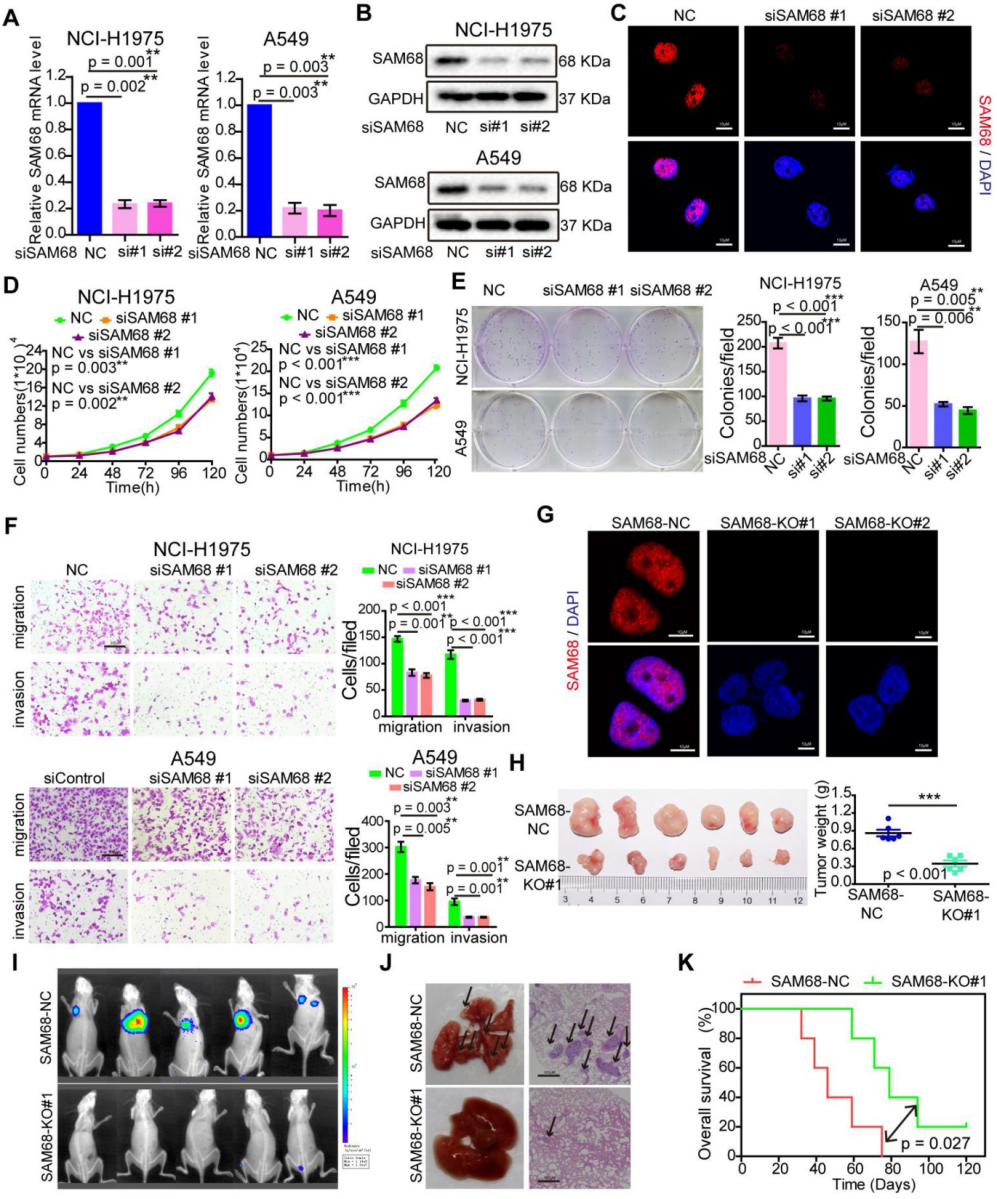
** Downregulation of SAM68 inhibited the malignant phenotypes of LUAD cells *in vitro* and tumorigenesis and progression *in vivo*.** (A and B) NCI-H1975 and A549 cells were transfected with anti-SAM68 siRNAs, *SAM68* mRNA (A) and protein levels (B) level were determined by qPCR and Western blot, respectively. (C) After SAM68 silencing, SAM68 was immuno-stained with anti-SAM68 antibody in NCI-H1975 cells. (D-F) The effects of SAM68 silencing on NCI-H1975 and A549 cells growth (D), colony formation (E), and migration and invasion (F) were detected. (G) CRISPR-Cas9 mediated knockout (KO) of *SAM68* in NCI-H1975 cells as detected by immunofluorescence. (H) The* in vivo* growth of *SAM68* KO (sgSAM68) NCI-H1975 cellswas detected (n = 6). (I)The *in vivo* lung metastasis of* SAM68* KO NCI-H1975 cells was examined (n = 5). (J and K) The hematoxylin and eosin (HE) staining and Kaplan-Meier curves are shown for two cohorts of transplanted mice carrying *SAM68* KO cells and control groups. Data are represented as mean ± SEM. *p < 0.05, **p < 0.01 or ***p < 0.001. Two-tailed t-tests were used A, E, F and I. Two-way ANOVA was used in D. Log rank tests was used in L.

To elucidate the effects of *SAM68* on cancer cell growth, colony formation, migration, and invasion, *SAM68* was overexpressed or silenced in LUAD cells (Figure 2A-C; see also Figure S1A-D). Cancer cell growth, colony formation, migration, and invasion significantly decreased when *SAM68* was silenced (Figure 2D-F) and increased when *SAM68* was ectopically expressed in the lung cancer cell lines NCI-H1975 and A549 (Figure S1E-G).

To determine the role of *SAM68* in tumorigenesis *in vivo*, we generated a *SAM68* knockout (KO) NCI-H1975 cell line by CRISPR-Cas9 technology (Figure 2G and Figure S2). As shown in Figure 2H, the *in vivo* growth of LUAD cell xenografts composed of *SAM68* KO NCI-H1975 cells was clearly impaired. Furthermore, the lungs of mice injected with luciferase-tagged *SAM68* KO NCI-H1975 cells developed metastatic nodules, whereas those with control NCI-H1975 cells did not (Figure 2I). Lung metastatic nodules were confirmed by histological analysis (Figure 2J). Kaplan-Meier survival analyses showed that mice with cancer xenografts carrying *SAM68* KO NCI-H1975 cells were at a lower risk of cancer-related death than were mice with cancer xenografts carrying control NCI-H1975 cells (Figure 2K, *p* = 0.027, log-rank test). These data provided direct evidence that downregulation of *SAM68* suppresses cancer cell growth, colony formation, migration, and invasion *in vitro* and tumorigenesis and metastasis *in vivo*.

### SAM68 interacts with hnRNP A1

**Figure 3:**
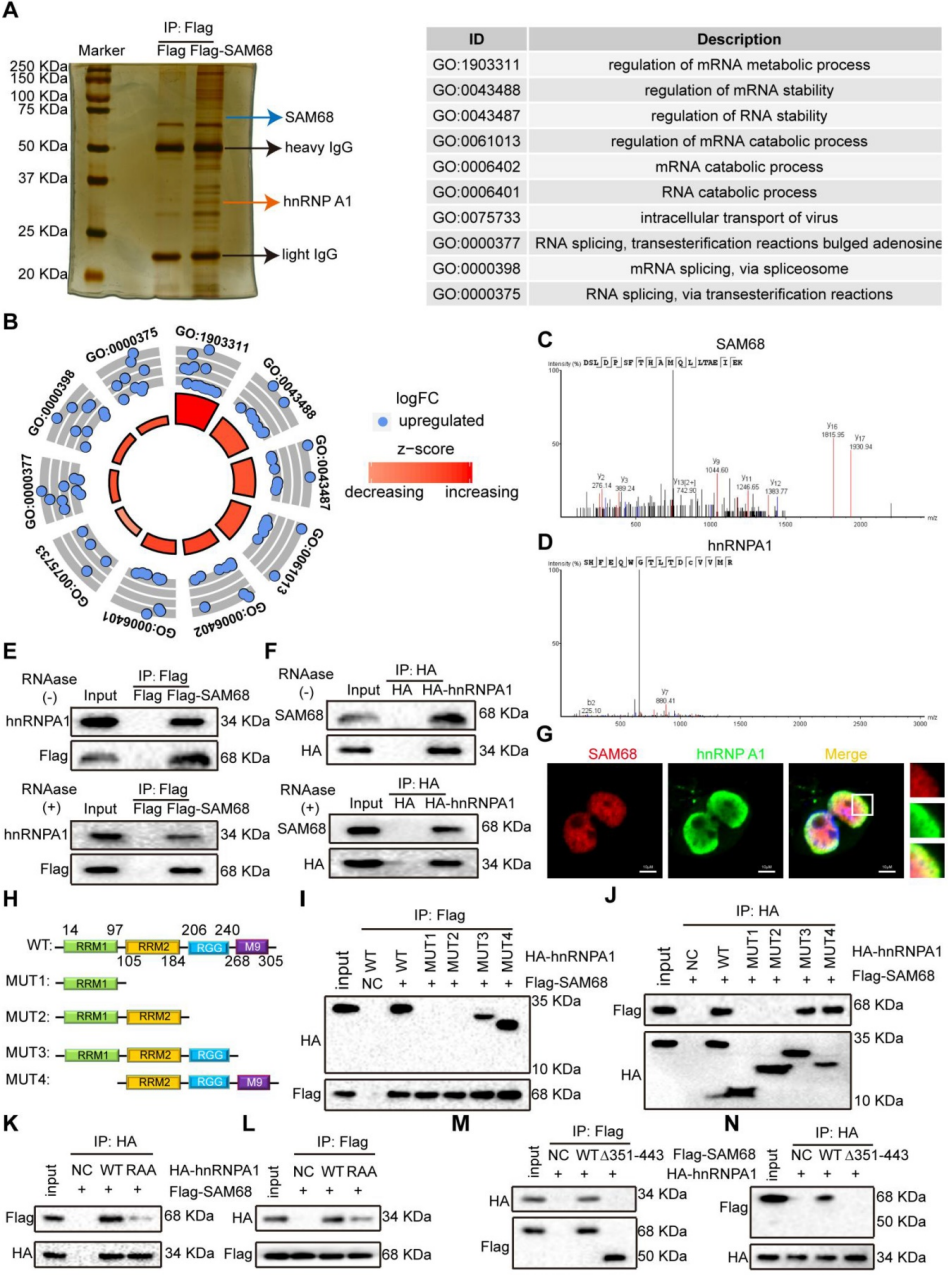
** The 351~443 motif of SAM68 binds to the Glycine residues in RGG box of hnRNP A1.** (A) HEK293T cells were transfected with Flag-SAM68. After Co-IP, proteins that interacted with Flag-SAM68 were identified using silver staining combined mass spectrometry. (B) The SAM68-interacting proteins were performed by GO analysis. (C and D) The unique peptide of SAM68 (C) and hnRNP (D) A1 were identified by mass spectrometry. (E and F) Flag-SAM68 and HA-hnRNP A1 plasmid were transfected into NCI-H1975 cells, cellular lysates were treated with 10 mg/mL RNase A (Thermofisher, EN0531) for 1 h or no treatment, Flag-SAM68 complexes were co-immunoprecipitated by anti-Flag antibody, then hnRNP A1 was detected (E), and HA-hnRNP A1 complexes were co-immunoprecipitated by anti-HA antibody, then SAM68 was detected (F). (G) Confocal images of endogenous SAM68 and hnRNP A1 in NCI-H1975 cells. (H) Wild-type (WT) and indicated mutations with the different domain of hnRNP A1 were constructed. (I and J) The indicated HA-hnRNP A1 WT and mutation constructs and Flag-SAM68 were co-transfected into HEK293T cells, Flag-SAM68 and HA-hnRNP A1 complexes were co-immunoprecipitated by anti-Flag and HA antibody, respectively; HA-hnRNP A1 mutants were detected using anti-HA antibodies, and Flag-SAM68 were detected using anti-Flag antibodies. (K and L) The HA-hnRNP A1 WT or its RAA mutation with Flag-SAM68 were co-transfected into HEK293T cells, and the interactions of hnRNP A1 RAA mutant with Flag-SAM68 were detected as described in (I) and (J). (M and N) The Flag-SAM68 WT or its indicated mutation with HA-hnRNP A1 were co-transfected into HEK293T cells, the interactions of Flag-SAM68 indicated mutation with HA-hnRNP A1 were detected as described in (I) and (J).

To further elucidate the mechanism by which SAM68 promotes cancer cell invasion and metastasis, coimmunoprecipitation (Co-IP) and mass spectrometry were performed to identify the proteins that interact with SAM68 (Figure 3A). We identified 39 proteins interacting with SAM68 (Table S2). Gene Ontology was used to characterize the functional roles of SAM68 and its interacting proteins. SAM68-interacting proteins were mainly enriched in RNA splicing (Figure 3B), suggesting that SAM68 could regulate cellular RNA splicing.

Among the 39 proteins that interacted with SAM68, hnRNP A1 had the highest protein score (Table S2). hnRNP A1 regulates the splicing of PKM mRNA, promoting the formation of PKM2. PKM2 is a key determinant of the metabolic phenotype in tumor cells that promotes aerobic glycolysis and provides favorable conditions for tumorigenesis. Therefore, we focused on the interaction between SAM68 and hnRNP A1. Unique peptides of SAM68 and hnRNP A1 were identified by mass spectrometry (Figure 3C-D). We further confirmed the interactions between SAM68 and hnRNP A1 by using co-IP with or without RNase A treatment (Figure 3E-F) and fluorescence co-localization (Figure 3G). We also confirmed that RNase A treatment did not affect coIP of the endogenous SAM68-hnRNP A1 complex (Figure S3A-B).

**Figure 4:**
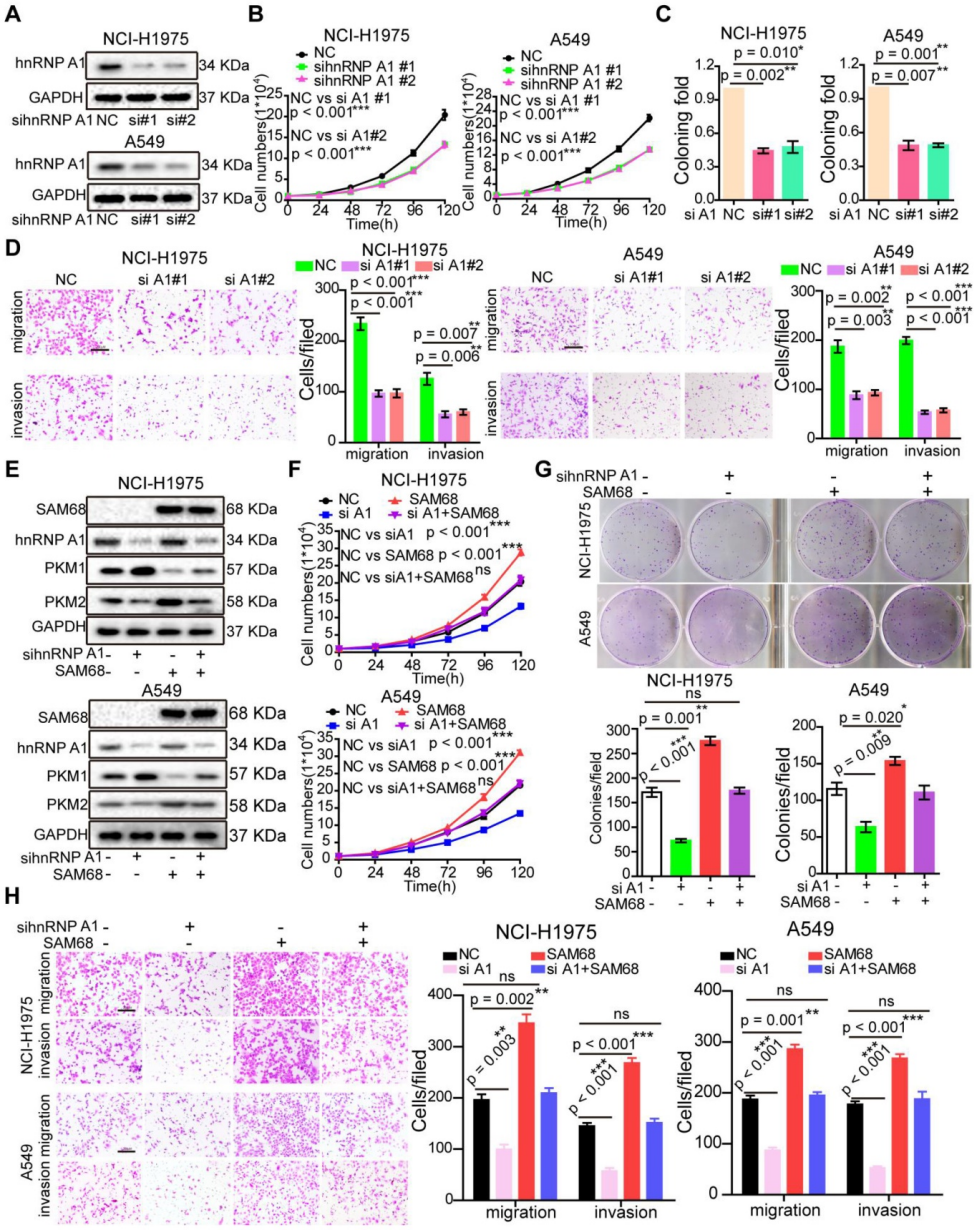
**Silencing hnRNP A1 antagonized the enhancement of malignant phenotypes induced by SAM68 overexpression.** (A~D) The anti-hnRNP A1 siRNAs were transfected into NCI-H1975 and A549 cells, hnRNP A1 protein levels (A) level was detected by Western blot, and the effects of silencing hnRNP A1 on NCI-H1975 and A549 cells growth (B), colony formation (C), migration and invasion (D) were detected. (E~H) The Flag-SAM68 plasmid and anti-hnRNPA1 siRNAs were co-transfected into NCI-H1975 and A549 cells, the indicated protein levels (E), cell growth (F), colony formation (G), migration and invasion (H) were detected. Data are represented as mean ± SEM. *p < 0.05, **p < 0.01 or ***p < 0.001. Two-way ANOVA were used in B and F, two-tailed t-tests were used C, D, G and H.

hnRNP A1 consists of two *N*-terminal RNA recognition motifs (RRM1 and RRM2), a C-terminal RNA-binding RGG box (RGG), and a nuclear targeting sequence (M9) 25 (Figure 3H). To dissect the hnRNP A1 domain required for interaction with SAM68, hnRNP A1 truncated fusion constructs with a C-terminal HA tag were constructed and co-expressed with the SAM68-Flag vector in HEK293T cells (Figure 4H). Only hnRNP A1 constructs containing the RGG motif bound to the SAM68, indicating that the RGG motif of hnRNP A1 is essential for SAM68 binding (Figures 3I-J).

The RGG box regulates the binding of proteins containing the RGG motif to RNA, and mediates the interaction of RGG motif proteins with other proteins 26,27. Glycine (G) residues are critical for binding because the interactions are charge-based 26,27. We therefore constructed an hnRNP A1 RAA mutant by changing the glycine residues (G) to alanine residues (A) in three RGGs in the RGG box of hnRNP A1. The mutation prevented the binding of SAM68 to hnRNP A1 (Figure 3K-L), indicating that the G residues in the RGG box of hnRNP A1 are essential for binding to SAM68. To determine which SAM68 domain binds to the RGG box of hnRNP A1, a truncated SAM68 fusion construct with a C-terminal Flag tag was constructed as described by Paronetto et al. 10. We confirmed that the 351-443 aa region of SAM68 interacted with hnRNP A1 (Figure 3M-N). Therefore, SAM68 interacts with hnRNP A1 via binding of the 351-443 aa region to the hnRNP A1 RGG motif.

### SAM68 promotes hnRNP A1-stimulated LUAD cell growth

To determine the role of hnRNP A1 in LUAD, we studied hnRNP A1 mRNA expression in lung tissues and LUAD tissues by using four microarray gene expression datasets deposited in the Oncomine, GEO, and TCGA databases. The *hnRNP A1* mRNA levels were higher in LUAD tissues than in normal tissues (Figure S4A-D), indicating that *hnRNP A1* expression is upregulated in LUAD. Interestingly, SAM68 and hnRNP A1 were upregulated in multiple identical lung cancer gene expression datasets, which is in accordance with our previous results.

As expected, hnRNP A1silencing suppressed cell growth, colony formation, migration, and invasion in NCI-H1975 and A549 LUAD cells (Figures 4A-D), which is consistent with the cancer phenotypes induced by SAM68 knockdown.

To determine whether SAM68 promotes tumorigenesis through hnRNP A1, the SAM68-Flag vector and anti-hnRNP A1siRNA were co-transfected into NCI-H1975 and A549 LUAD cells (Figure 4E). hnRNP A1knockdown completely abolished the SAM68 overexpression-induced increases in cell proliferation, colony formation, migration, and invasion ability, suggesting that SAM68 promotes tumorigenesis through hnRNP A1 (Figure 4F-H). Collectively, our data indicate that SAM68 promotes tumorigenesis by binding to hnRNP A1.

### SAM68 increases hnRNP A1 binding to the sequences flanking the *PKM* exon 9 by binding to the RGG box of hnRNP A1

To further study the mechanism underlying the effects of SAM68 on lung cancer tumorigenesis, we performed high-throughput RNA-seq of WT and SAM68-overexpressing NCI-H1975 cells.

**Figure 5:**
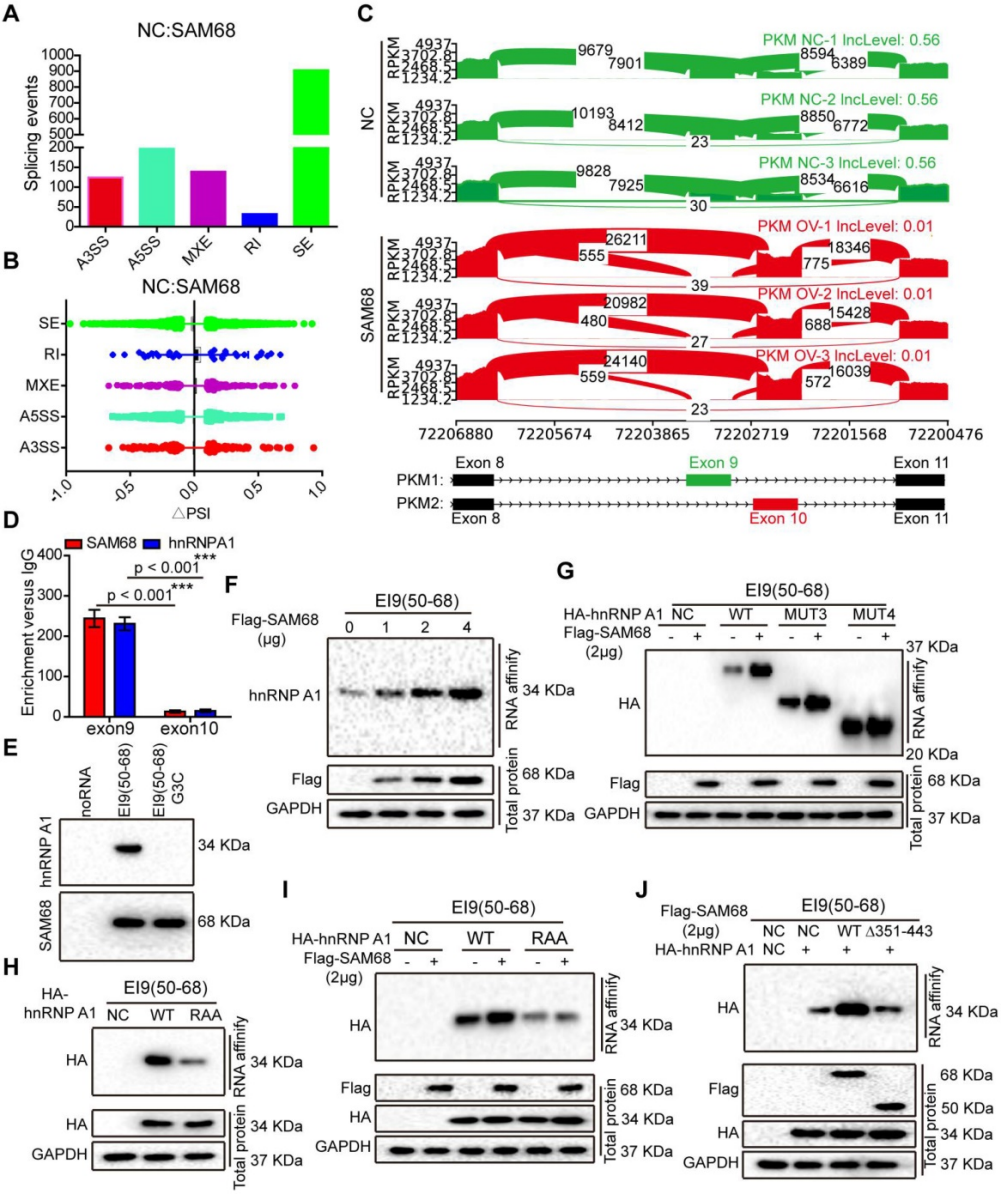
** The 351~443 motif of SAM68 favors the binding of the RGG motif of hnRNP A1 to the intronic sequences flanking exon 9 (EI9) by binding to the Glycine residues in RGG box of hnRNP A1.** (A) Quantification of SAM68-regulated AS events in each category was measured RNA sequencing. (A3SS/A5SS, alternative 3'/5' splice sites, MXE, mutually exclusive exons, RI, retained introns, SE, skipped exons) (B) Changes in PSI values of SAM68-regulated AS events were shown. (C) SAM68 regulated PKM pre-mRNA splicing and promoted PKM2 isoform formation. (D) CLIP assay of SAM68 and hnRNP A1 binding to the PKM pre-mRNA. NCI-H1975 cells were UV-crosslinked and immunoprecipitated with control IgGs or antibodies, as indicated. (E) RNA affinity purification followed by Western blot showed *in vitro* binding of the indicated biotin-labeled RNAs with endogenous hnRNP A1 or SAM68. (F) The Flag-SAM68 plasmid at the indicated doses was transfected into NCI-H1975 cells, and RNA affinity purification was performed using biotin-labeled RNA EI9 (50-68). (G) NCI-H1975 cells were co-transfected with Flag-SAM68 plasmid and the indicated hnRNP A1 mutations, and *in vitro* binding of EI9 (50-68) RNA probes with WT or the indicated hnRNP A1 mutations was detected. (H) The HA-hnRNP A1 WT or its RAA mutation was transfected into NCI-H1975 cells, and RNA affinity purification was performed using biotin-labeled RNA EI9 (50-68). (I) HA-hnRNP A1 WT or its RAA mutation with Flag-SAM68 plasmids were co-transfected into NCI-H1975 cells, and *in vitro* binding of EI9 (50-68) RNA probes with WT or its RAA mutation was detected. (J) The WT SAM68 or its indicated mutant with WT hnRNP A1-HA plasmids were cotransfected into NCI-H1975 cells, and RNA affinity purification was performed using biotin-labeled RNA EI9 (50-68).

By using a ∆PSI cutoff of 0.1 and FDR < 0.05, we identified 1398 SAM68-regulated AS events (Table S3 and GSE149120). Among different types of AS events, including alternative 3ʹ/5ʹ splice site (A3SS/A5SS), mutually exclusive exon (MXE), retained intron, and skipped exon, SAM68-overexpressing cells preferentially induced exon skipping (Figures 5A-B). Based on the FDR value, RPKM, and ∆PSI> 0.15, we selected FN1, ZNF621, ACADVL, OSBPL1A, and ANAPC11 to verify the reliability of the RNA-seq analysis. SAM68 regulates FN1, ZNF621, ACADVL, OSBPL1A, and ANAPC11 pre-mRNAs splicing (Figure S5A-K). Among the 1398 SAM68-regulated AS events, it is particularly interesting that SAM68 regulates pre-mRNA PKM splicing, ultimately promoting PKM2 formation while inhibiting PKM1 formation (Figure 5C). Further, hnRNP A1 represses the inclusion of the PKM flanking exon 9 by binding of the hnRNP A1 RGG box to the intronic UAGGGC sequences flanking exon 9, thereby inhibiting E9 inclusion and contributing to the formation of the PKM2 isoform 23,28. SAM68 did not alter the hnRNP A1 protein level (Figure S6). To test whether SAM68 and hnRNP A1 bind PKM pre-mRNA, we performed UV crosslink immunoprecipitation (CLIP) experiments. SAM68 and hnRNP A1 showed strong binding at exon 9 with PKM pre-mRNA, and weak binding to exon 10 (Figure 5D). Therefore, we speculated that SAM68 interacts with A1 to form a splicing complex to regulate PKM mRNA splicing.

To determine the effect of SAM68 on the ability of hnRNP A1 to bind to PKM E9, we performed RNA affinity chromatography by using 5ʹ biotin-labeled RNA corresponding to EI9 (50-68) containing the UAGGGC sequence EI9 (50-68) G3C (mutation of the G3 nucleotide to C in UAGGGC), as previously described 29. hnRNP A1 and SAM68 directly bound to the EI9 (50-68) sequence of *PKM* (Figure 5E). SAM68 expression markedly increased the binding ability of hnRNP A1 to the EI9 sequences of *PKM*, and the enhancement effects were dose-dependent (Figure 5F), suggesting that both proteins cooperate in the regulation of PKM splicing.

We then demonstrated that SAM68 expression enhanced binding of the hnRNP A1 RGG box to the EI9 sequence of *PKM* (Figure 5G). The hnRNP A1 RGG box binds to the EI9 sequence of *PKM* 25, which is consistent with our result of the hnRNP A1 RAA mutant not binding to the EI9 sequence of *PKM* (Figure 5H), indicating that the G residues in the RGG box of hnRNP A1 are essential for hnRNP A1 binding to the EI9 sequence of *PKM*. Moreover, SAM68 failed to promote binding of the mutant hnRNP A1 to the EI9 sequence of *PKM* (Figure 5I), suggesting that SAM68 increases the binding ability of hnRNP A1 to the EI9 sequence of *PKM* in a G-residue-dependent manner. Our results showed that SAM68 interacts with hnRNP A1 via the binding of the 351-443 aa region to the RGG motif of hnRNP A1 (Figure 3M-N). SAM68 did not induce hnRNP A1 binding to the EI9 sequence of *PKM* when the 351-443 aa region of SAM68 was mutated (Figure 5J). Collectively, our data suggest that SAM68 increases hnRNP A1 binding to the sequences flanking PKM exon 9 by binding of the 351-443 aa region of SAM68 to the RGG box of hnRNP A1.

### SAM68 drives hnRNP A1-dependent PKM splicing and PKM2 isoform formation

**Figure 6:**
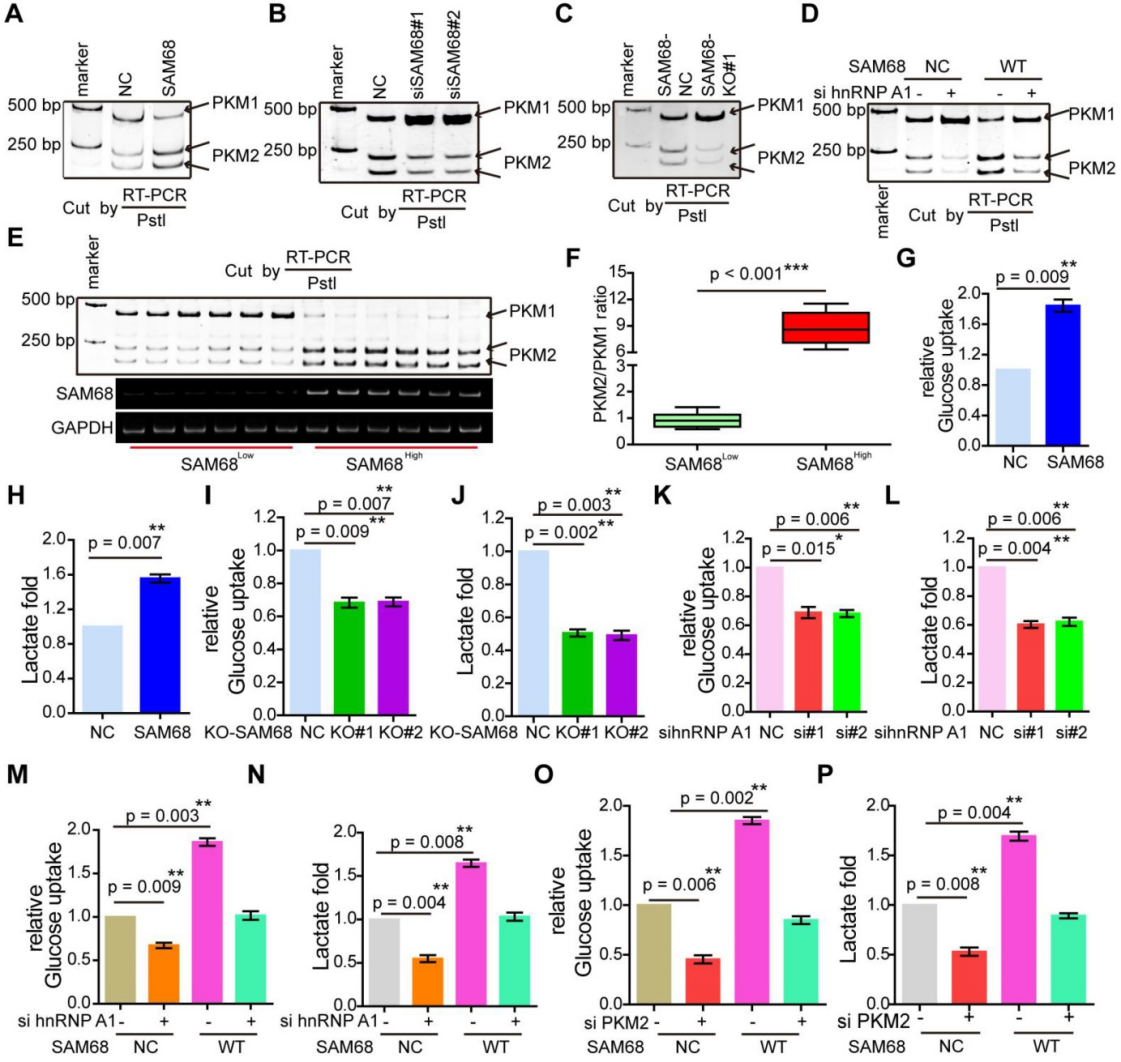
** Sam68 and hnRNP A1 cooperated in *PKM* splicing to decrease PKM1 isoform formation and increase PKM2 isoform formation, then promoting the aerobic glycolysis of LUAD cells.** (A and B) NCI-H1975 cells were transfected with the Flag-SAM68 plasmids (A) or anti-SAM68 siRNAs (B), followed by the PKM splicing assay. (C) *PKM* splicing was performed in *SAM68* KO NCI-H1975 cells. (D) The Flag-SAM68 plasmid and anti-hnRNPA1 siRNAs were co-transfected into NCI-H1975, followed by the PKM splicing assay. (E) *PKM* splicing was performed in the LUAD tissue samples with low (n = 6) and high (n = 6) SAM68 expression. (F) The ratio of PKM2/PKM1 is calculated in the LUAD tissue samples with low (n = 6) and high (n = 6) SAM68 expression (G and H) NCI-H1975 cells were transfected with the Flag-SAM68 plasmids, glucose uptake and lactate production were measured. (I and J) Glucose uptake and lactate production were measured in *SAM68* KO NCI-H1975 cells. (K and L) NCI-H1975 cells were transfected with anti- hnRNP A1 siRNAs, then glucose uptake (K) and lactate production (L) were detected. (M and N) The Flag-SAM68 plasmid and anti-hnRNPA1 siRNAs were co-transfected into NCI-H1975, and glucose uptake (M) and lactate production (N) were detected. (O and P) The Flag-SAM68 plasmid and anti-PKM2 siRNAs were co-transfected into NCI-H1975, and glucose uptake (O) and lactate production (P) were detected.

hnRNP A1 represses the inclusion of PKM E9 by binding to the E19 sequence of *PKM*, promoting PKM2 isoform formation 29. Our data indicate that SAM68 promotes the binding of hnRNP A1 to EI9 of the *PKM* gene. Thus, we analyzed the effect of SAM68 on *PKM* splicing by RT-PCR and restriction digestion to assess the PKM1 and PKM2 levels, as previously described 30. SAM68 overexpression increased the *PKM2* mRNA and protein levels and decreased *PKM1* mRNA and protein levels (Figure 6A and Figure 5D), and SAM68 knockdown or knockout exhibited the opposite effect (Figure 6B-C).

To further determine whether SAM68 promotes the stimulatory effects of hnRNP A1 on PKM2 formation, we co-transfected NCI-H1975 cells with SAM68-Flag and anti-hnRNP A1. The SAM68 overexpression-induced enhancement of the PKM2 isoform and reduction of the PKM1 isoform were completely attenuated by hnRNP A1 silencing (Figure 6D), suggesting that SAM68-mediated PKM2 isoform formation and inhibition of PKM1 isoform formation is hnRNP A1-dependent.

We then studied PKM1 isoform and PKM2 isoform expression in tissue samples with high or low SAM68 expression (Figure 6E). The PKM2/PKM1 ratio was significantly higher in tissue samples with high SAM68 expression than in those with low SAM68 expression (Figure 6F). We also analyzed the PKM1 and PKM2 mRNA levels in six pairs of matched fresh normal and high SAM68 expression LUAD tissues, and found higher PKM2 mRNA levels and lower PKM1 mRNA levels in LUAD tissues compared to those in normal tissues (Figure S7A). The *PKM2* and *PKM1* mRNA levels were positively (R = 0.671,* p* = 0.0011) and negatively (R = -0.699, *p* = 0.0007) correlated, respectively, with SAM68 protein levels in LUAD tissue samples (Figure S7B). Thus, our results indicate that SAM68 drives hnRNP A1-dependent *PKM* splicing and PKM2 isoform formation.

### SAM68 promotes tumorigenesis via PKM2

Considering that SAM68 drives hnRNP A1-dependent PKM splicing and PKM2 formation, we then determined whether SAM68 stimulates LUAD cell tumorigenesis through PKM2. SAM68-Flag vector and anti-PKM2 siRNAs were co-transfected into NCI-H1975 and A549 LUAD cells (Figure S8A). SAM68 overexpression-induced enhancement in cell proliferation, colony formation, and migration, and invasion was completely attenuated by PKM2 silencing (Figure S8B-D). Therefore, SAM68 stimulates LUAD cell tumorigenesis mainly through PKM2.

### SAM68 promotes hnRNP A1-dependent aerobic glycolysis

PKM2 plays a key role in the determination of aerobic glycolysis or the Warburg effect with increased glucose uptake and lactate production 21,22. To determine whether SAM68, which decreases PKM1 isoform and increases PKM2 isoform levels, is sufficient to drive cancer metabolic reprogramming, we measured glucose uptake and lactate production in NCI-H1975 LUAD cells. SAM68 increased the glucose uptake and lactate production (Figure 6G-H), and SAM68 silencing decreased glucose uptake and lactate production (Figure 6I-J) in cancer cells, suggesting that SAM68 promoted aerobic glycolysis. Further, hnRNP A1 knockdown decreased cell glucose uptake and lactate production (Figure 6K-L), which is consistent with the effects of SAM68 knockdown. SAM68 overexpression enhanced glucose uptake and lactate production, which were completely blocked by hnRNP A1 silencing (Figure 6M-N). As expected, PKM2 knockdown decreased glucose uptake and lactate production (lanes 1 and 2 in Figure 6O-P). The SAM68-induced increase in glucose uptake and lactate production were completely attenuated by PKM2 silencing (Figure 6O-P). These results suggested that SAM68 promotes aerobic glycolysis in LUAD cells by inducing hnRNP A1-dependent PKM splicing and PKM2 isoform formation.

### SAM68 promotes PKM splicing and tumorigenesis by binding to hnRNP A1

**Figure 7:**
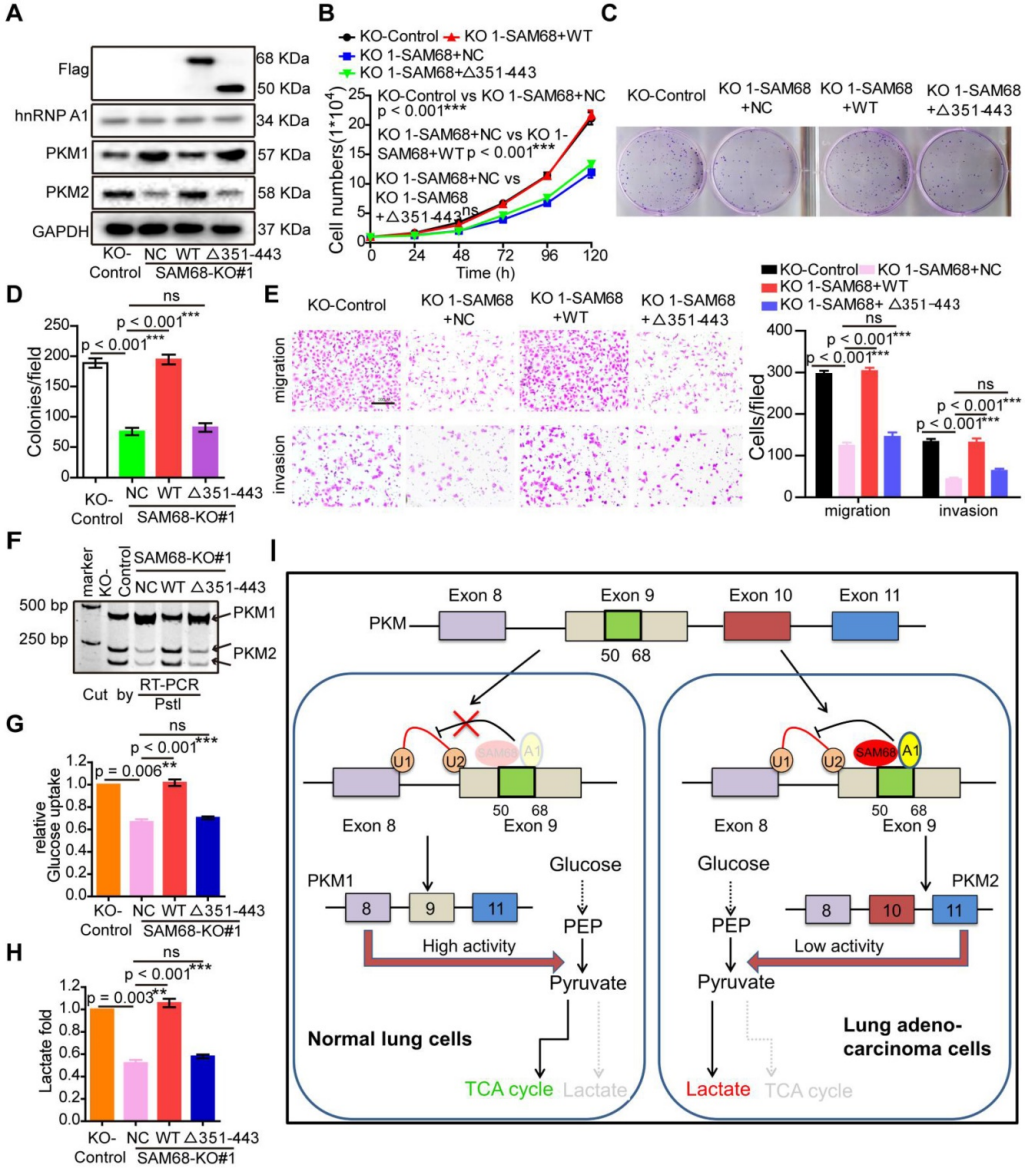
** SAM68 increases PKM2 isoform formation, and promotes malignant phenotypes and aerobic glycolysis by cooperating to hnRNP A1**. The WT SAM68 or its indicated mutant was transfected into *SAM68* KO NCI-H1975 cells; the indicated proteins were detected by Western blot (A), and cell growth (B), colony formation (C and D), migration and invasion (E), PKM splicing (F), glucose uptake (G), and lactate production (H) were detected. (I) A regulatory model of SAM68 on tumorigenesis proposed in this study. Data are represented as mean ± SEM. **p < 0.01 or ***p < 0.001. Two-way ANOVA was used in B; two-tailed t-tests were used in D~ H.

SAM68 increases binding of the hnRNP A1 RGG box to *PKM* exon 9, inducing PKM2 isoform formation via binding of the 351-443 aa region of SAM68 to the RGG motif of hnRNP A1. We therefore determined whether SAM68-mediated tumorigenesis, PKM splicing, and aerobic glycolysis is hnRNP A1 binding-dependent. Expression of wild-type (WT) SAM68 or binding-defective SAM68Δ351-443 mutant was restored in SAM68 knockout cells (Figure 7A). SAM68 silencing caused a significant decrease in cell growth, colony formation, migration, and invasion, which could all be restored to the control levels after supplementing with WT SAM68 but not with hnRNP A1 binding-defective SAM68 Δ351-443 mutant (Figure 7B-E and 7G-H). Furthermore, SAM68 Δ351-443 mutant did not induce the inclusion of *PKM* exon 9 to promote PKM2 isoform formation (Figure 7F). Thus, SAM68 promotes tumorigenesis, *PKM* splicing, and aerobic glycolysis mainly via its interaction with hnRNP A1.

## Discussion

SAM68 was previously identified as the first specific target of the Src tyrosine kinase in mitosis 4,5. We demonstrated that human SAM68 expression is significantly higher in LUAD tissues than in adjacent non-tumor lung tissues. *SAM68* mRNA levels were higher in LUAD tissues than in normal lung tissues in TCGA datasets deposited in the Oncomine database, indicating that *SAM68* upregulation in LUAD is induced at the transcriptional level. *SAM68* upregulation was significantly associated with aggressive LUAD phenotypes, and LUAD patients with SAM68^high^ exhibited higher death rates and shorter survival times than did LUAD patients with SAM68^low^. Our data suggest that SAM68 could be a novel independent prognostic factor in patients with LUAD. Downregulation of *SAM68* significantly inhibited malignant LUAD phenotypes* in vitro* and tumorigenesis and metastasis* in vivo,* suggesting that SAM68 could also find application as a novel therapeutic target for LUAD.

SAM68 promotes aerobic glycolysis by regulating PKM2 alternative splicing in colorectal cancer 31. In this study, we observed SAM68-mediated regulation of PKM alternative splicing and metabolic reprogramming in LUAD. Thus, SAM68 likely regulates PKM splicing-mediated reprogramming in many tumor types. SAM68 mRNA and protein levels were higher in primary LUAD tissues than in corresponding nontumor tissues, and SAM68 could serve as an independent prognostic factor in patients with LUAD. We found that SAM68 promotes tumorigenesis, and elucidated the novel mechanism underlying the effect of SAM68 on cancer progression. Binding of the 351-443 aa region of SAM68 to the RGG motif of hnRNP A1 could increase hnRNP A1 binding to exon 9 to repress E9 in the PKM transcript, contributing to increased PKM2 formation and decreased PKM1 formation. Previous studies have shown that the C-terminal region of SAM68 (351-443) interacts with hnRNPA1, collectively regulating the alternative splicing of Bcl-x in HEK293 cells 10. In addition, the C-terminal region of SAM68 (351-443) interacts with hnRNPA1, collectively regulating SMN2 exon-7 skipping in SMA cells 11. The mechanism of splicing regulation by SAM68 and hnRNPA1 proposed in these studies is very similar to that indicated by our results. However, we confirmed for the first time that SAM68 interacts with the hnRNPA1 RGG box.

HnRNPA1, hnRNPA2/B1, and PTBP1 regulate PKM splicing, enhancing the expression of the PKM2 isoform and suppressing that of the PKM1 isoform 23,24. We determined that hnRNPA1 and hnRNPA2/B1 interact with SAM68. We used overexpression of hnRNPA1 or hnRNPA2/B1 in SAM68-KO LUAD cells to determine the effect of SAM68 on hnRNPA1 and hnRNPA2/B1-dependent regulation of PKM splicing. The hnRNPA1 overexpression-induced increase in PKM2/PKM1 ratio was completely attenuated by SAM68 depletion (Figure S9). However, SAM68 expression deficiency did not completely attenuate hnRNPA2/B1 overexpression-induced increase of PKM2/PKM1 ratio (Figure S9). HnRNPA2/B1, an m6A reader, has a key role in RNA processing via m6A modification of pre-mRNA or pre-miRNA 32,33. We speculate that hnRNPA2/B1 acts as a splicing regulator, and may also regulate PKM splicing via other pathways, such as m6A modification.

SAM68 promotes tumorigenesis by regulating NF-κB pathway-dependent anti-apoptotic transcription and AKT/GSK-3*β*/Snail pathway-dependent epithelial-mesenchymal transition in colon and cervical cancers, respectively 13,34. In this study, we determined the role of SAM68 in LUAD, and elucidated a novel mechanism by which SAM68 mediates LUAD invasion and metastasis. Our data showed that SAM68 promotes metastasis and invasion in LUAD by interacting with hnRNP A1. SAM68 increases the binding of hnRNP A1 to exon 9 of the *PKM* gene to repress E9 in the PKM transcript by binding of the 351-443 aa region of SAM68 to the RGG motif of hnRNP A1, thereby promoting PKM2 formation and inhibiting PKM1 formation, consequently promoting cancer metastasis and invasion. AKT2 increased *PKM2* mRNA levels in ovarian cancer cells 35. SAM68 functions in conjunction with the oncogene metadherin to regulate alternative splicing of proteins involved in prostate cancer progression 36. These results are similar to our findings that SAM68 operates in conjunction with hnRNP A1 to regulate PKM alternative splicing. In this study, we discovered a novel mechanism by which a SAM68-mediated increase in PKM2 drives hnRNP A1-dependent PKM splicing. PKM2 expression mediates metabolic advantages in cancer cells and is a critical determinant of aerobic glycolysis 22. To our knowledge, this is the first study to report that SAM68 promotes aerobic glycolysis in an hnRNP A1-dependent manner.

## Conclusions

In summary, we found that SAM68 promotes LUAD cell tumorigenesis and cancer metabolic programming by binding of the 351-443 aa region of SAM68 to the RGG motif of hnRNP A1, driving hnRNP A1-dependent *PKM* splicing, contributing to increased PKM2 isoform formation and inhibition of PKM1 isoform formation. *SAM68* mRNA and protein levels were upregulated in primary LUAD cancer tissues. LUAD patients with SAM68^high^ exhibited higher death rates and shorter survival times than LUAD patients with SAM68^low^. Our findings indicate that SAM68 may be a new independent prognostic factor and potential therapeutic target for patients with LUAD.

## Materials and Methods

### Cells and cell cultures

NCI-H1975 cells (ATCC) and A549 cells (ATCC) were cultured in RPMI 1640 (Gibco, C11875500BT), and Human HEK293T cells (ATCC) were cultured in DMEM (Gibco, 11995065). All cells were supplemented with 10% FBS (Gibco, 10270106) and 1% penicillin and streptomycin (Thermo Fisher Scientific, 15140122), and cultured at 37 °C with 5% CO_2_.

### Patients, tissue samples and immunohistochemistry

LUAD tissues and paired adjacent normal lung tissues were collected from LUAD patients at the Sixth Affiliated Hospital of Guangzhou Medical University. These samples did not receive any preoperative anti-cancer treatment and had definite pathological diagnosis. Tissue microarray chips containing 50 LUAD tissue samples and 50 paired adjacent normal lung tissue were purchased from Alenabio (Xi'an, China). Immunohistochemistry assays were performed as previously described 25. In short, after dewaxing, the tissue microarray chip was treated with 3% hydrogen peroxide in methanol and blocked with a standard labeled streptavidin biotin kit (DAKO, Germany), incubated overnight with SAM68 polyclonal antibody (proteintech, 10222-1-AP, 1:100) in a moist chamber at 4 °C. Next day, after incubated with biotinylated goat anti-rabbit antibody for 1 h, the tissue microarray chip was stained with DAKO liquid 3,3'-diaminobenzidine tetrahydrochloride (DAB). According to the staining intensity (no staining, 0; mild staining, 1; moderate staining, 2; and severe staining, 3) and the number of positive cells (≤ 10% positive cells, 0; 10-30% positive cells, 1;31-50% positive cells, 2; 51-80% positive cells, 3; and >80% positive cells, 4), each specimen was scored. The final score for each case is the sum of the score for the percentage of positive cells and the score for staining intensity.

### Western blotting

Tissue samples or Cells samples were lysed using SDS lysis buffer (beyotime, P0013G). All samples were separated on 10-15% SDS PAGE and polyvinylidene fluoride (PVDF) membrane. After incubated using blocking buffer (6% skim milk in PBS with 0.1% Tween 20), the blots were incubated with primary antibodies. After washing three times with 0.1% PBST (PBS with 0.1% Tween 20), the blots were incubated with a horseradish peroxidase-conjugated (HRP) secondary antibody and detected with Supersignal West Pico (Thermo Fisher Scientific, 34080). The primary and Secondary antibodies used in our study are listed in the Table S1.

### RT-PCR and qRT-PCR

The total RNA of tissue samples and cells samples was obtained using the TRIzol lysis buffer (Invitrogen, 10296010). cDNA was obtained using a PrimeScript^TM^ RT reagent kit with gDNA Eraser (TaKaRa, RR037A). The SYBR Premix Ex Taq™ II kit was used to conduct qRT-PCR. The agarose gel electrophoresis with polymerase Chain reaction was used to conduct RT-PCR. The primers used in this study are shown in the Table S1.

### Plasmid constructs and transfection

The SAM68-Flag or hnRNP A1-HA fusion protein constructs was cloned into the pcDNA3.1(+) vector by Clon Express MultiS One Step Cloning Kit (Vazyme, C113-01) according to the manufacturer's protocol. The mutants of hnRNP A1-HA (hnRNP A1^mut1~4^-HA and hnRNP A1^RAAmut^-HA) were produced by Mut Express II Fast Mutagenesis Kit V2 (vazyme, C214). The primers of plasmid constructs are shown in the Table S1. Plasmid constructs were transfected into cells with Lipofectamine™ 2000 Transfection Reagent (Invitrogen, 11668027). SiRNA against SAM68, hnRNP A1 or PKM2 were produced in GenePharma. The siRNAs were transfected into cells with Lipofectamine RNA iMAX (Invitrogen, 13778075). The siRNAs used in this study are shown in the Table S1.

### SAM68 KO cell lines by CRISPR-Cas9

The gRNA sequences of knocking out (KO) SAM68 were designed using the SYNTHEGO online tool (https://design.synthego.com), are shown in the Table S1. Human NCI-H1975 cells were transiently transfected with a constructed pSpCas9(BB)-2A-GFP plasmids (Addgene, #48138) containing SAM68 sgRNAs using Lipofectamine™ 2000 Transfection Reagent (Invitrogen, 11668027). After transfection for 48h, single cells were planted into 96-well plates. After four weeks, independent clones were harvested and was tested by Western blotting with anti-SAM68 antibodies. In addition, gDNA of independent clones were extracted and tested by RT-PCR. The primers are shown in the Table S1.

### Immunofluorescence assay

The constructed plasmid or indicated siRNAs were transfected into LUAD cells for 24h. LUAD Cells were plated on coverslips for 24h. After fixed with 4% paraformaldehyde for 20 min at room temperature, Cells were permeabilizated using 0.1% Triton X-100 for 7 min. Next, after blocked with 2% BSA in PBS for 2h, Cells were incubated with primary antibody overnight at 4 °C (1:500 for SAM68 and hnRNP A1). After 3 times wash with 0.1% PBST, secondary goat anti-rabbit cy3 (beyotime, A0428, 1:500 dilution) or goat anti-mouse AlexaFluor 488 (beyotime, A0502, 1:500 dilution) were added for 2 h incubation. After 3 times wash with 0.1% PBST, coverslips were stained with DAPI (beyotime, C1005).

### Cell growth and colony formation assay

The constructed plasmid or indicated siRNAs were transfected into LUAD cells for 36h. For cell growth, NCI-H1975 cells and A549 cells were seeded in 24-well plates at 10000 cells/well. The cell number in each treatment group was counted at 24, 48, 72, 96, and 120 h. For colony formation, 400 NCI-H1975 cells and A549 cells were seeded in 6-well plates and cultured for 7 days. After fixed with anhydrous methanol, the plates were stained using 1% crystal violet solution.

### Migration and invasion assays

The migration and invasion assays* in vitro* were performed using Transwell chambers (Becton, 353504). The constructed plasmid or indicated siRNAs were transfected into LUAD cells for 48h. For migration assays, 5 × 10^4^ NCI-H1975 cells and A549 cells in 200 μl starvation media were seeded into the top chambers of transwell chamber and cultured for 18h, while RPMI 1640 culture media with 10% FBS was applied on the bottom. For invasion assays, the upper transwell chambers were coated with matrigel (Corning, 356234). 1 × 10^5^ NCI-H1975 cells and A549 cells in 200 μl starvation media were seeded into the upper transwell chambers and cultured for 36h, while RPMI 1640 culture media with 10% FBS was applied on the bottom. After fixed with anhydrous methanol, migrated and invaded cells were stained with 1% crystal violet, captured and counted under a 10× microscope.

### Animal breeding and treatments

Four-week-old female BALB/c mice were purchased from Guangdong Medical Laboratory Animal Center (Foshan, China). Four-week-old female NOD/SCID mice were purchased from Charles River Laboratories (Beijing, China). All mice were housed in laminar flow cabinets under specific pathogen-free (SPF) conditions. The animal experiments were approved by the Laboratory Animal Ethics Committee of the Sixth Affiliate Hospital of Guangzhou Medical University. For tumor growth assay *in vivo*, a total of 6 × 10^6^ NCI-H1975-NC and NCI-H1975-SAM68-KO cells were subcutaneously injected into Left and right armpits of forelimbs in BALB/c mice, respectively (n = 6). After 4 weeks, the mice were killed by suffocation with carbon dioxide, and the tumors were dissected and weighed. For lung metastasis assay, 1 × 10^6^ NCI-H1975-luc-NC and NCI-H1975-SAM68-KO cells were injected into NOD/SCID mice through the tail vein. After implantation for 10 weeks, the metastatic foci in the lungs were was quantified by D-luciferin injection and IVIS spectrum imaging (PerkinElmer).

### Co-immunoprecipitation and mass spectrometry

Cells grown at 70-80% confluency were transfected with the indicated plasmid for 48h. Cells were lysed in IP lysis buffer (beyotime, P0013) adding 1x phosphatase inhibitor cocktail (Sigma-Aldrich & Roche). After lysed on ice for 30 min, lysates were separated by centrifuge at 13,000 rpm for 10 min, 4 °C. Pierce™ BCA protein assay kit (thermofisher, 23227) were used to quantify the protein concentrations. Equal amounts of proteins were immunoprecipitated with indicated antibody for 12h. After 12h, proteins were incubated with Protein A/G PLUS-Agarose (Santa cruz, sc-2003) for 4h. After washed 3 times with ice-cold PBS, proteins were eluded with using SDS lysis buffer (beyotime, P0013G). The samples were separated by 10% SDS PAGE gel. The gel was stained using the Fast Silver Stain Kit (beyotime, P0017S) according to the manufacturer's protocol. Differential protein bands were detected by nano-LC-MS/MS (AB SCIEX TripleTOF 5600, USA). Proteins were identified by the Mascot program against the Uniprot human protein database.

### RNA-seq and analysis

Three biological replicates for SAM68 Over-expression or control cells were harvested in 1 ml TRIzol. RNAs were extracted using the TRIzol Reagent kit from Invitrogen. All samples were sequenced by Illumina Novaseq6000 of Novogene Company (Beijing, China) with a paired-end 150 bp read length. Data filtering was performed with the following protocol: (1) remove reads with adaptor sequences; (2) remove reads in which the percentage of unknown bases (N) is more than 10%; (3) remove low quality reads in which the base with a quality value ≤ 5 is more than 50% in a read. Fastp (default parameters) was used for assessed RNA-seq reads quality and remove potentially remained adaptor sequences or poor quality reads 37. First 18 bases of the reads are trimmed due to GC bias on the head of reads. After quality control, clean reads were mapped to GRCh38 human reference (ftp://ftp.ncbi.nlm.nih.gov/genomes/all/GCA/000/001/405/GCA_000001405.15_GRCh38/seqs_for_alignment_pipelines.ucsc_ids/GCA_000001405.15_GRCh38_no_alt_analysis_set.fna.gz) sequence by using hisat2 (hisat2 -p 10 --dta -x GCA_000001405.15_GRCh38_no_alt_analysis_set -1 sample.1.fastq.gz -2 sample.2.fastq.gz | samtools sort -@ 10 -o sample.bam) 38,39. Alternative splicing events were analyzed using the rMATS program on hisat2 output bam file 40. Alternative splicing events plots are generated by rmats2sashimiplot (https://github.com/Xinglab/rmats2sashimiplot). RNA-seq data in this study have been deposited into the GEO database (https://www.ncbi.nlm.nih.gov/geo/query/acc.cgi?acc=GSE149120).

### UV-crosslinking immunoprecipitation

According to results of previous studies, the CLIP analysis was performed 41,42. After irradiated on ice (100 mJ/cm^2^), treated cells were lysed using lysis buffer (50 mM Tris, pH 8.0, 100 mM NaCl, 1 mM MgCl_2_, 0.5 mM Na_3_VO_4_, 0.1 mM CaCl_2_, 1% NP-40, 1 mM DTT, RNase inhibitor (Takara, 2313A), and protease inhibitor cocktail (Roche, 5892970001). Next, after briefly sonicated on ice, cell lysis samples were incubated with DNase (Ambion, AM2222) at 37 °C for 10 min and then centrifuged at 13 000g at 4 °C for 5 min. After diluted to 1 ml with lysis buffer, 1 mg of supernatant extract was incubated with rabbit anti-SAM68, mouse anti-hnRNP A1 or IgGs (negative control) for immunoprecipitation in the presence of protein A/G PLUS-Agarose (Santa cruz; sc-2003). Next, 1 mg of supernatant extract was incubated with 1000 IU RNase I (Ambion, AM2294) and rotated at 4 °C for 2 h. After stringent washes, an aliquot (10%) was used to keep as a control of immunoprecipitation, while the rest was incubated with proteinase K (Invitrogen, AM2546) at 55 °C for 30 min. The RNA was purified by TRIzol method, reversely transcripted and detected by q-PCR.

### RT-PCR and PKM splicing assays

As previously described 23, PKM splicing assays were conducted. The total RNA of tissue samples and cells samples was obtained using the TRIzol lysis buffer (Invitrogen, 10296010). cDNA was obtained using a PrimeScript^TM^ RT reagent kit with gDNA Eraser (TaKaRa, RR037A). The PCR products of PKM were digested with PstI endonuclease (New England Biolabs, R0140S) and were resolved by 8% non-denaturing PAGE. The primers of PKM are shown in the Table S1.

### RNA affinity purification

As previously described 43, the RNA affinity purification was performed. The 5'biotin-labeled PKM EI9 RNAs were synthesized by Genscript company (Nanjing, China) according to Chen et al.'s research 29. 1 nmol biotin-labeled RNAs was bound with 50 μl Streptavidin-Agarose beads (Sigma, GE17-5113-01) to prepare RNA-immobilized beads. Furthermore, cells grown at 70-80% confluency were transfected with the indicated plasmid for 48h. Cells were lysed in IP lysis buffer (beyotime, P0013) adding 1× phosphatase inhibitor cocktail (Sigma-Aldrich & Roche). Nuclear pellets were collected with Nuclear and Cytoplasmic Protein Extraction Kit, and lysed by sonication. Nuclear lysates were incubated with RNA-immobilized beads at 30 °C for 30 min while rotating. After protein and RNA binding, proteins were eluded with using SDS lysis buffer (beyotime, P0013G). The samples were detected by Western blotting.

### Measurement of glucose uptake and lactate production

The constructed plasmid or indicated siRNAs were transfected into LUAD cells for 48h. The glucose concentration in cultured media was detected using Glucose Colorimetric Assay kit (BioVision, K606-100) following the manufacturer's instructions. The lactate production in cultured media was measured using the Lactate Colorimetric Assay kit II (BioVision, K627-100) following the manufacturer's instructions.

### Statistics

Data was reported as mean ± SD from three independent experiments. Data was analyzed by two-tailed Student's t-test between two groups. Kaplan-Meier curve with the log-rank test was applied to perform the survival analysis. Graph Pad Prism 5.0 software was used to statistical analysis. NS, no significant, *p < 0.05, **p < 0.01, ***p < 0.001.

## Abbreviations

SAM68: SRC-associated in mitosis, 68 kDa
LUAD: lung adenocarcinoma
PKM: pyruvate kinase
hnRNPs: heterogeneous nuclear ribonucleoproteins
siRNAs: small interfering RNAs
qRT-PCR: quantitative reverse transcription-PCR
GEO: Gene expression omnibus

## References

[1] Zappa C, Mousa SA (2016). Non-small cell lung cancer: current treatment and future advances. Transl Lung Cancer Res.

[2] Siegel RL, Miller KD, Jemal A (2019). Cancer statistics, 2019. CA Cancer J Clin.

[3] Zhang N, Liang R, Gensheimer MF, Guo M, Zhu H, Yu J (2020). Early response evaluation using primary tumor and nodal imaging features to predict progression-free survival of locally advanced non-small cell lung cancer. Theranostics.

[4] Fumagalli S, Totty NF, Hsuan JJ, Courtneidge SA (1994). A target for Src in mitosis. Nature.

[5] Taylor SJ, Shalloway D (1994). An RNA-binding protein associated with Src through its SH2 and SH3 domains in mitosis. Nature.

[6] Bielli P, Busà R, Paronetto MP, Sette C (2011). The RNA-binding protein Sam68 is a multifunctional player in human cancer. Endocr Relat Cancer.

[7] Busà R, Geremia R, Sette C (2010). Genotoxic stress causes the accumulation of the splicing regulator Sam68 in nuclear foci of transcriptionally active chromatin. Nucleic Acids Res.

[8] Matter N, Herrlich P, König H (2002). Signal-dependent regulation of splicing via phosphorylation of Sam68. Nature.

[9] Valacca C, Bonomi S, Buratti E, Pedrotti S, Baralle FE, Sette C (2010). Sam68 regulates EMT through alternative splicing-activated nonsense-mediated mRNA decay of the SF2/ASF proto-oncogene. J Cell Biol.

[10] Paronetto MP, Achsel T, Massiello A, Chalfant CE, Sette C (2007). The RNA-binding protein Sam68 modulates the alternative splicing of Bcl-x. J Cell Biol.

[11] Pedrotti S, Bielli P, Paronetto MP, Ciccosanti F, Fimia GM, Stamm S (2010). The splicing regulator Sam68 binds to a novel exonic splicing silencer and functions in SMN2 alternative splicing in spinal muscular atrophy. Embo J.

[12] Song L, Wang L, Li Y, Xiong H, Wu J, Li J (2010). Sam68 up-regulation correlates with, and its down-regulation inhibits, proliferation and tumourigenicity of breast cancer cells. J Pathol.

[13] Li Z, Yu CP, Zhong Y, Liu TJ, Huang QD, Zhao XH (2012). Sam68 expression and cytoplasmic localization is correlated with lymph node metastasis as well as prognosis in patients with early-stage cervical cancer. Ann Oncol.

[14] Mascarenhas JB, Tchourbanov AY, Danilov SM, Zhou T, Wang T, Garcia J (2018). The Splicing Factor hnRNPA1 Regulates Alternate Splicing of the MYLK Gene. Am J Respir Cell Mol Biol.

[15] Michael WM, Choi M, Dreyfuss G (1995). A nuclear export signal in hnRNP A1: a signal-mediated, temperature-dependent nuclear protein export pathway. Cell.

[16] Gao X, Wan Z, Wei M, Dong Y, Zhao Y, Chen X (2019). Chronic myelogenous leukemia cells remodel the bone marrow niche via exosome-mediated transfer of miR-320. Theranostics.

[17] Geuens T, Bouhy D, Timmerman V (2016). The hnRNP family: insights into their role in health and disease. Hum Genet.

[18] Park SJ, Lee H, Jo DS, Jo YK, Shin JH, Kim HB (2015). Heterogeneous nuclear ribonucleoprotein A1 post-transcriptionally regulates Drp1 expression in neuroblastoma cells. BBA-gene Regul Mech.

[19] Jean-Philippe J, Paz S, Caputi M (2013). hnRNP A1: the Swiss army knife of gene expression. Int J Mol Sci.

[20] Koppenol WH, Bounds PL, Dang CV (2011). Otto Warburg's contributions to current concepts of cancer metabolism. Nat Rev Cancer.

[21] Israelsen WJ, Vander HM (2015). Pyruvate kinase: Function, regulation and role in cancer. Semin Cell Dev Biol.

[22] Christofk HR, Vander HM, Harris MH, Ramanathan A, Gerszten RE, Wei R (2008). The M2 splice isoform of pyruvate kinase is important for cancer metabolism and tumour growth. Nature.

[23] David CJ, Chen M, Assanah M, Canoll P, Manley JL (2010). HnRNP proteins controlled by c-Myc deregulate pyruvate kinase mRNA splicing in cancer. Nature.

[24] Calabretta S, Bielli P, Passacantilli I, Pilozzi E, Fendrich V, Capurso G (2016). Modulation of PKM alternative splicing by PTBP1 promotes gemcitabine resistance in pancreatic cancer cells. Oncogene.

[25] Huang JZ, Chen M (2017). A Peptide Encoded by a Putative lncRNA HOXB-AS3 Suppresses Colon Cancer Growth. Mol Cell.

[26] Thandapani P, O'Connor TR, Bailey TL, Richard S (2013). Defining the RGG/RG Motif. Mol Cell.

[27] Chong PA, Vernon RM, Forman-Kay JD (2018). RGG/RG Motif Regions in RNA Binding and Phase Separation. J Mol Biol.

[28] Chen M, Zhang J, Manley JL (2010). Turning on a fuel switch of cancer: hnRNP proteins regulate alternative splicing of pyruvate kinase mRNA. Cancer Res.

[29] Chen M, David CJ, Manley JL (2012). Concentration-dependent control of pyruvate kinase M mutually exclusive splicing by hnRNP proteins. Nat Struct Mol Biol.

[30] Clower CV, Chatterjee D, Wang Z, Cantley LC, Vander HM, Krainer AR (2010). The alternative splicing repressors hnRNP A1/A2 and PTB influence pyruvate kinase isoform expression and cell metabolism. Proc Nat Acad Sci U S A.

[31] Zhao J, Li J, Hassan W, Xu D, Wang X, Huang Z (2020). Sam68 promotes aerobic glycolysis in colorectal cancer by regulating PKM2 alternative splicing. Ann Transl Med.

[32] Alarcón CR, Goodarzi H, Lee H, Liu X, Tavazoie S, Tavazoie SF (2015). HNRNPA2B1 Is a Mediator of m(6)A-Dependent Nuclear RNA Processing Events. Cell.

[33] Kwon J, Jo YJ, Namgoong S, Kim NH (2019). Functional roles of hnRNPA2/B1 regulated by METTL3 in mammalian embryonic development. Sci Rep.

[34] Fu K, Sun X, Wier EM, Hodgson A, Liu Y, Sears CL (2016). Sam68/KHDRBS1 is critical for colon tumorigenesis by regulating genotoxic stress-induced NF-κB activation. Elife.

[35] Zheng B, Geng L, Zeng L, Liu F, Huang Q (2018). AKT2 contributes to increase ovarian cancer cell migration and invasion through the AKT2-PKM2-STAT3/NF-kappaB axis. Cell Signal.

[36] Luxton HJ, Simpson BS, Mills IG, Brindle NR, Ahmed Z, Stavrinides V (2019). The Oncogene Metadherin Interacts with the Known Splicing Proteins YTHDC1, Sam68 and T-STAR and Plays a Novel Role in Alternative mRNA Splicing. Cancers (Basel).

[37] Chen S, Zhou Y, Chen Y, Gu J (2018). fastp: an ultra-fast all-in-one FASTQ preprocessor. Bioinformatics.

[38] Kim D, Paggi JM, Park C, Bennett C, Salzberg SL (2019). Graph-based genome alignment and genotyping with HISAT2 and HISAT-genotype. Nat Biotechnol.

[39] Li H, Handsaker B, Wysoker A, Fennell T, Ruan J, Homer N (2009). The Sequence Alignment/Map format and SAMtools. Bioinformatics.

[40] Shen S, Park JW, Huang J, Dittmar KA, Lu ZX, Zhou Q (2012). MATS: a Bayesian framework for flexible detection of differential alternative splicing from RNA-Seq data. Nucleic Acids Res.

[41] Bielli P, Sette C (2017). Analysis of *in vivo* Interaction between RNA Binding Proteins and Their RNA Targets by UV Cross-linking and Immunoprecipitation (CLIP) Method. Bio Protoc.

[42] Pagliarini V, Jolly A, Bielli P, Di Rosa V, De la Grange P, Sette C (2020). Sam68 binds Alu-rich introns in SMN and promotes pre-mRNA circularization. Nucleic Acids Res.

[43] Kashima T, Rao N, David CJ, Manley JL (2007). hnRNP A1 functions with specificity in repression of SMN2 exon 7 splicing. Hum Mol Genet.

