# Supplementary material for: SAM68 promotes tumorigenesis in lung adenocarcinoma by regulating metabolic conversion via PKM alternative splicing
Source: Theranostics. 2021 Jan 19;11(7):3359–75. doi: 10.7150/thno.51360 (PMC7847678; doi:10.7150/thno.51360)
Supplement: Supplementary file 1 — Supplementary figures and tables. [file thnov11p3359s1.zip › Supplementary material/Supplementary figures.pdf]

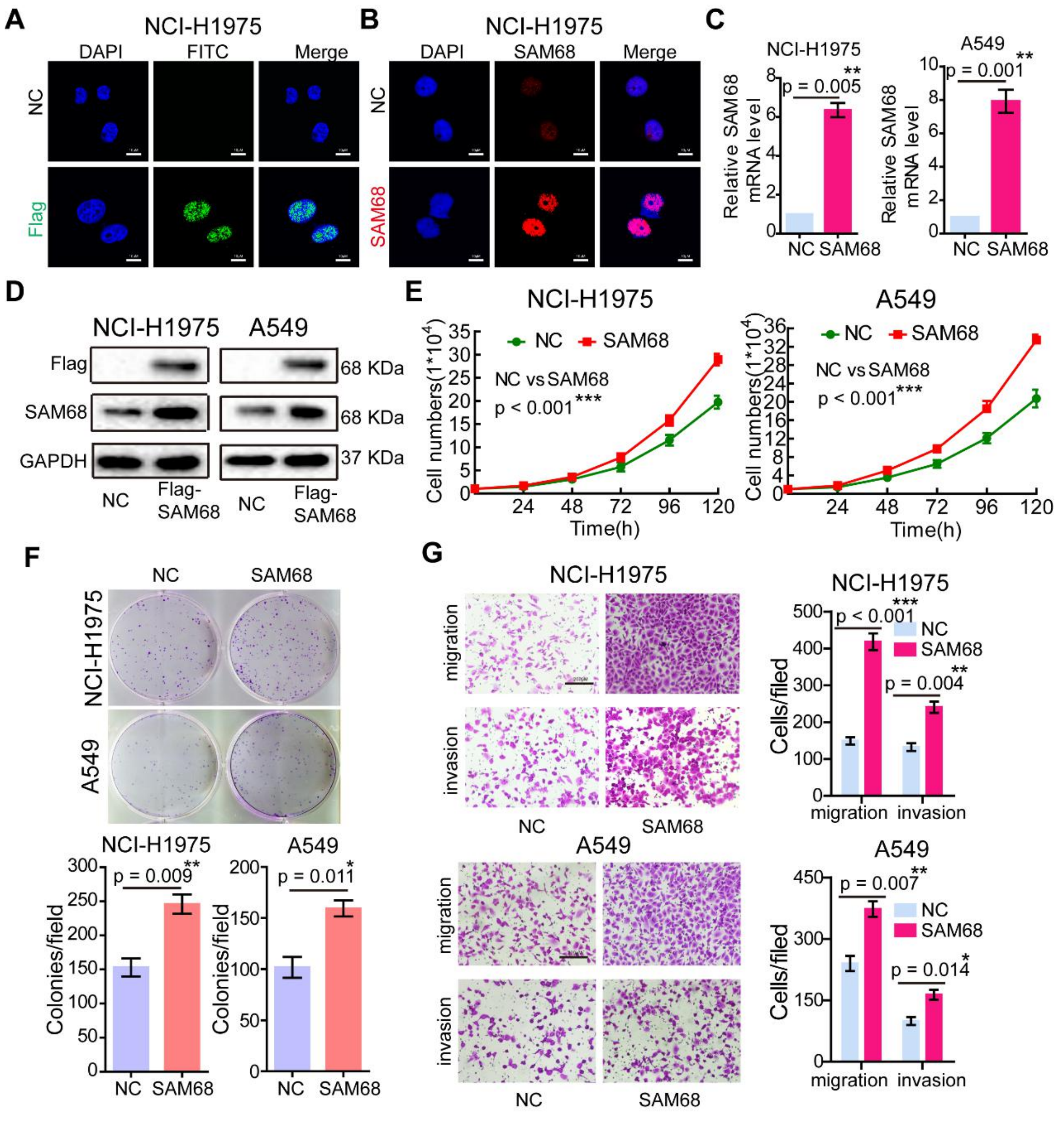

Figure S1 **overexpression of SAM68 promoted the malignant phenotypes of LUAD cells in vitro.** (A and B) NCI-H1975 cells were transfected with Flag-SAM68 plasmids, Flag (A) and SAM68 (B) were immuno-stained using anti-Flag and anti-SAM68 antibodies, respectively. (C and D) After SAM68 overexpression, *SAM68* mRNA (C) and protein levels (D) level were determined by qPCR and Western blot, respectively. (E~G) The effects of SAM68 overexpression on NCI-H1975 and A549 cells growth (E), colony formation (F), and migration and invasion (G) were detected. Data are represented as mean ±

SEM. *$p < 0.05$, **$p < 0.01$ or ***$p < 0.001$. Two-tailed t-tests were used C, F and G. Two-way ANOVA was used in E.

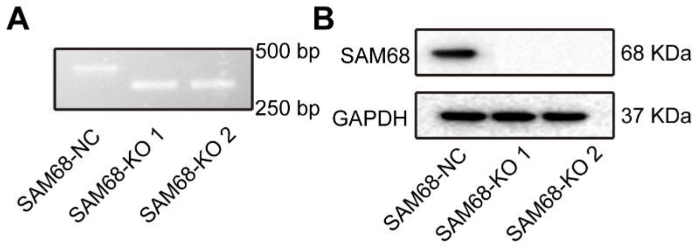

Figure S2 The SAM68 KO cell colonies were identified by RT-PCR (A) and Western blot (B).

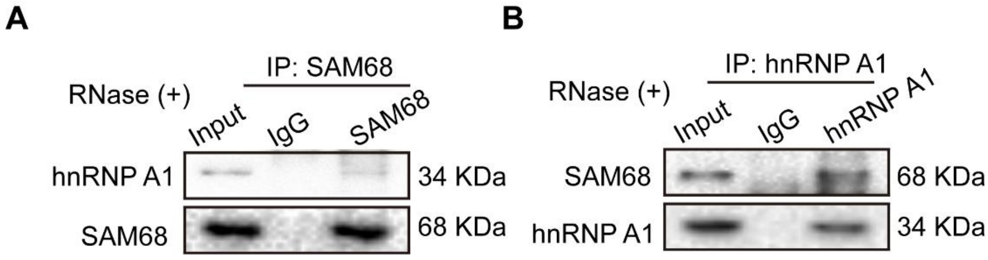

Figure S3 (A and B) Endogenous SAM68 and hnRNP A1 were coimmunoprecipitated using anti-SAM68 antibody or anti-hnRNP A1 antibody, in the presence of RNase A treatment.

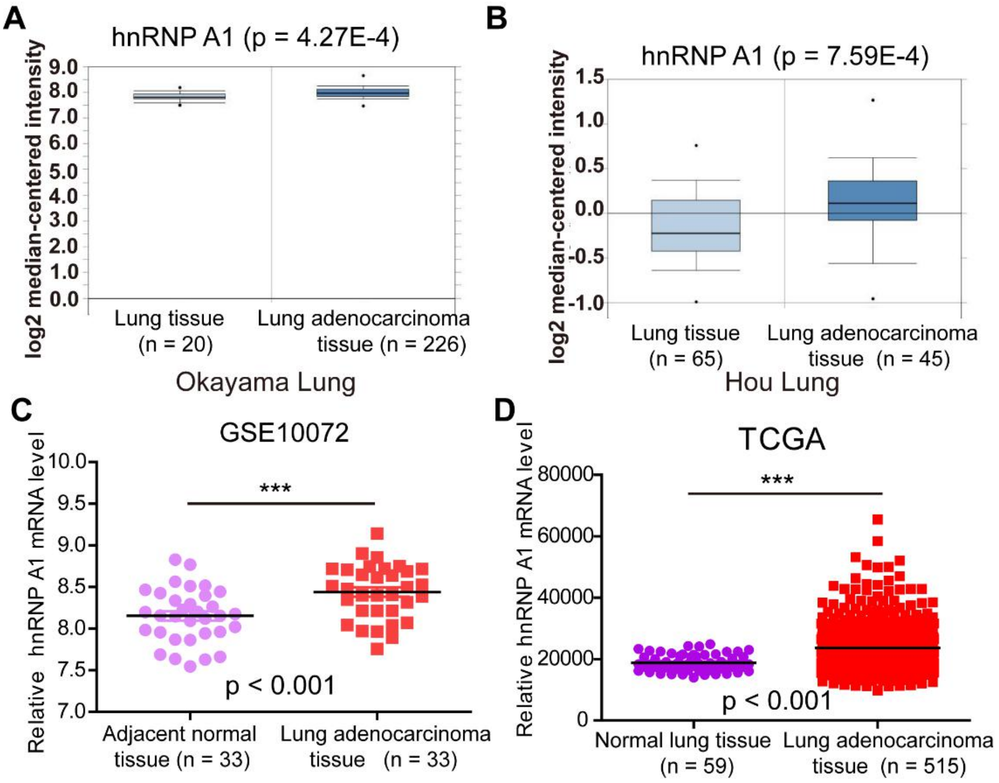

Figure S4 **hnRNP A1 is up-regulated in Lung adenocarcinoma (LUAD)** (A~D) *hnRNP A1* mRNA levels was up-regulated in LUAD compared to normal lung tissue based on the Oncomine (A and B), GEO (C) and TCGA (D) database.

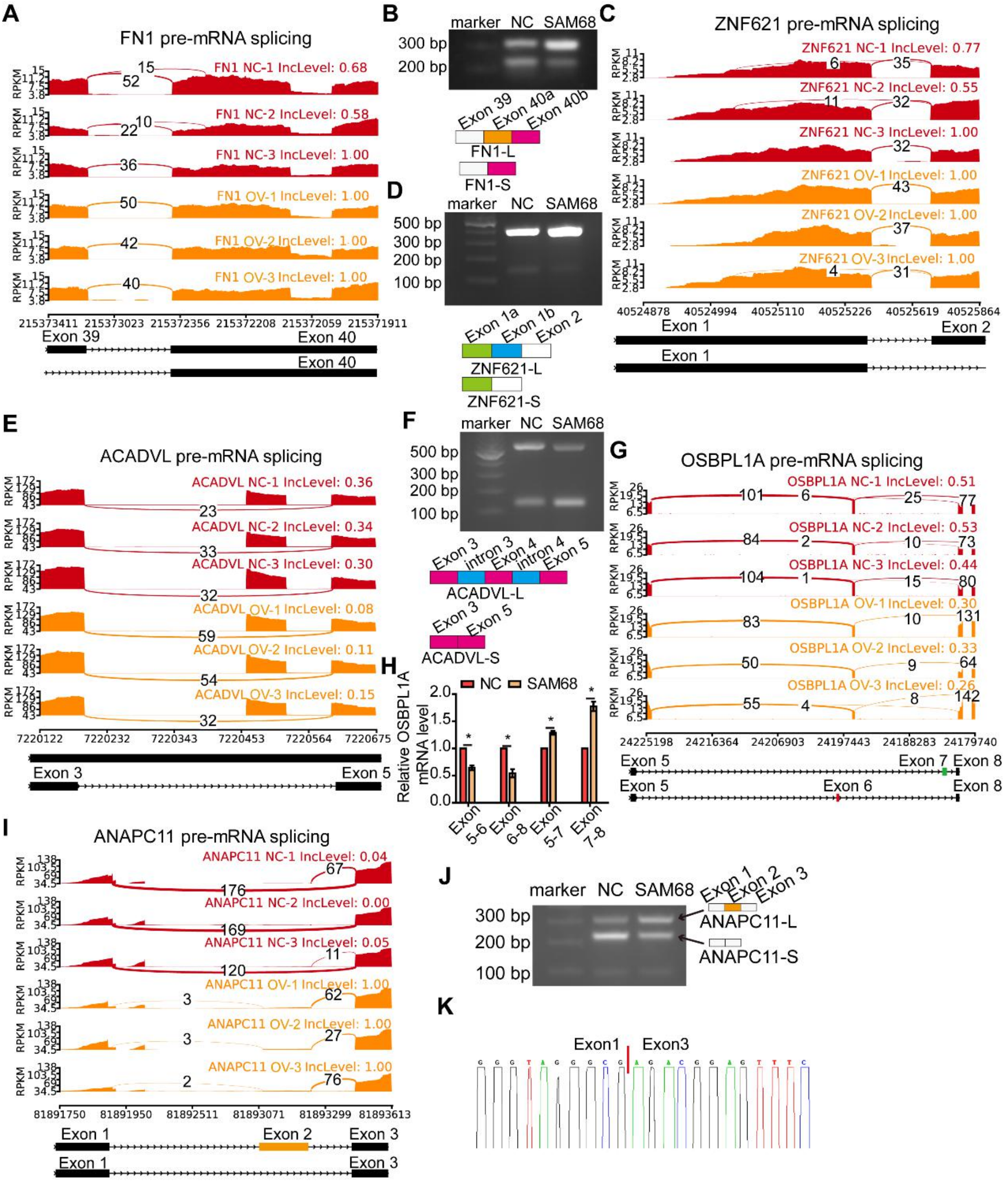

Figure S5 Five genes FN1, ZNF621, ACADVL, OSBPL1A and ANAPC11 were selected to verify reliability of the RNA-seq analysis (A) RNA-seq analysis showed that SAM68 regulated FN1 pre-mRNA splicing. (B) RT-PCR was used to detect the role of SAM68 in the regulation of FN1 exon40 alternative

splicing. (C) RNA-seq analysis showed that ZNF621 regulated ANAPC11 pre-mRNA splicing. (D) RT-PCR was used to detect the role of SAM68 in the regulation of ZNF621 exon1 alternative splicing. (E) RNA-seq analysis showed that SAM68 regulated ACADVL pre-mRNA splicing. (F) SAM68 promotes the inclusion of ACADVL intron3, exon 4 and intron 4 using RT-PCR. (G) RNA-seq analysis showed that SAM68 regulated OSBPL1A pre-mRNA splicing. (H) Q-PCR was used to detect the role of SAM68 in the regulation of OSBPL1A alternative splicing. (I) RNA-seq analysis showed that SAM68 regulated ANAPC11 pre-mRNA splicing. (J and K) SAM68 promotes the inclusion of ANAPC11 exon 2 using RT-PCR (J) and PCR fragment sequencing analysis(K).

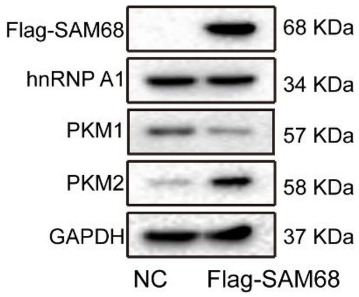

Figure S6 The Flag-SAM68 plasmid was transfected into NCI-H1975 cells, the indicated protein levels were detected.

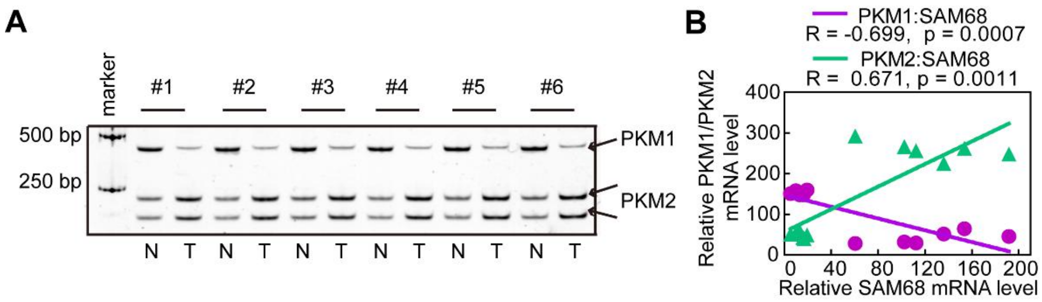

Figure S7 (A) *PKM* splicing was performed in the adjacent normal tissues and LUAD tissue samples with high SAM68 expression (n=6). (B) The PKM1 and PKM2 mRNA levels were negatively and positively correlated with *SAM68* mRNA levels in six pairs of matched LUAD tissues and corresponding normal lung tissues, respectively.

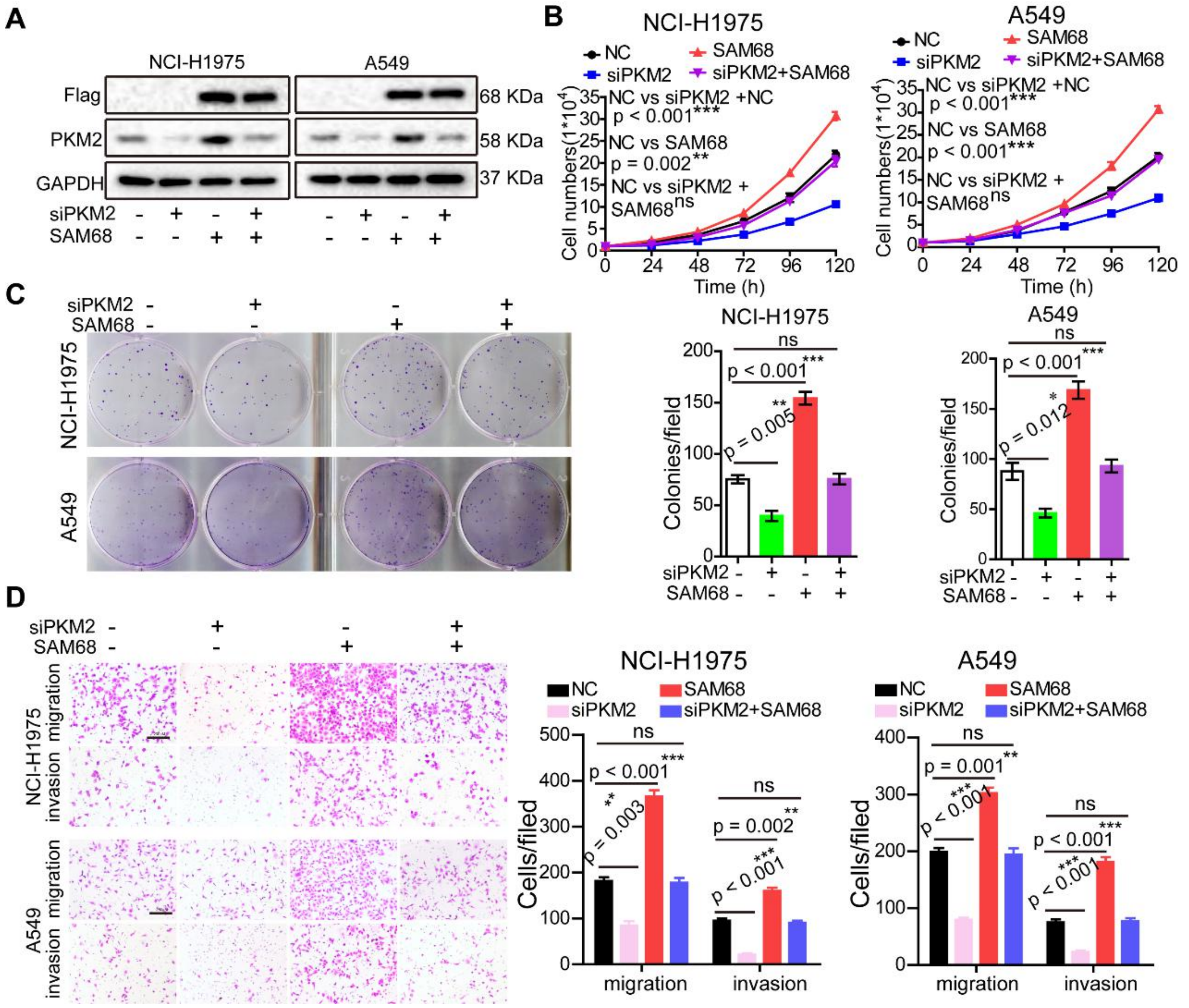

Figure S8. **Silencing PKM2 antagonized the enhancement of malignant phenotypes induced by SAM68 overexpression.** (A~B) The Flag-SAM68 plasmid and anti-PKM2 siRNAs were co-transfected into NCI-H1975 and A549 cells, the indicated protein levels (A), cell growth (B), colony formation (C), migration and invasion (D) were detected. Data arerepresented as mean ± SEM. *p < 0.05, **p < 0.01 or ***p < 0.001. Two-way ANOVA was used in B, two-tailed t-tests were used C and D.

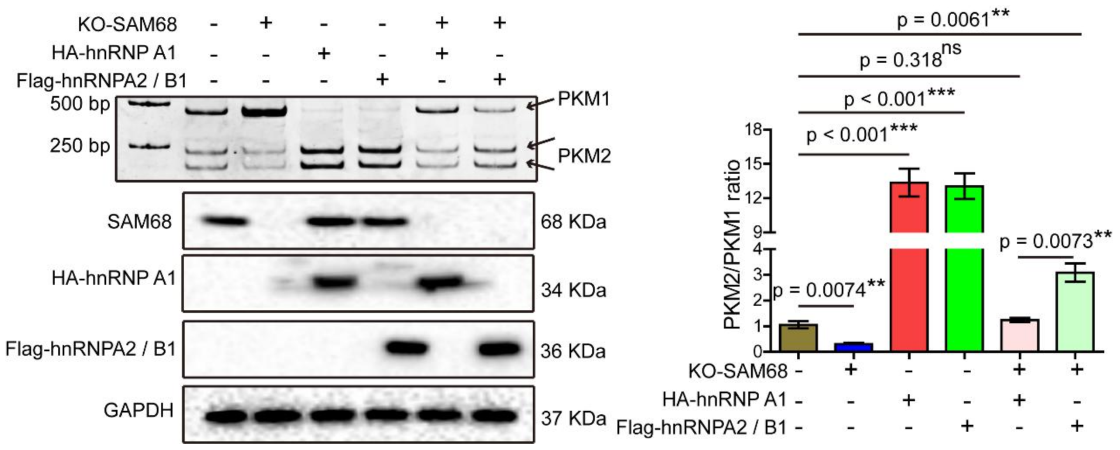

Figure S9. The hnRNPA1 or hnRNPA2/B1 plasmid was transfected into SAM68 KO NCI-H1975 cells, and the PKM splicing were detected.

Table S1. The antibodies, primers ,oligonucleotides and Cas9/sgRNAs used in this study are shown.		
**Antibodies**		
Rabbit Polyclonal anti-SAM68	Proteintech	Cat# 10222-1-AP
Mouse Monoclonal anti- hnRNP A1	Santa Cruz Biotechnology	Cat# sc-32301
Rabbit Polyclonal anti-PKM1	Proteintech	Cat# 15821-1-AP
Rabbit Polyclonal anti-PKM2	Proteintech	Cat#15822-1-AP
Rabbit Polyclonal anti-Flag	abcam	Cat# ab1162
Rabbit Polyclonal anti-HA	abcam	Cat# ab9110
Goat Anti-Mouse IgG (H+L) HRP	Proteintech	Cat# SA00001-1
Goat anti-Rabbit IgG (H+L) HRP	Proteintech	Cat# SA00001-2
Rabbit Polyclonal anti-GAPDH	Proteintech	Cat# 10494-1-AP
**Primers name**		
SAM68 (RT-PCR)	Forward	TTAACGGCAGTAGGCACCAC
	Reverse	GGCTAGATTTCAAAACCCACGTA
GAPDH (RT-PCR)	Forward	TCTTCCAGGAGCGAGATCCCT
	Reverse	TGGTCATGAGTCCTTCCACGAT
SAM68 (q-PCR)	Forward	ATTAACGGCAGTAGGCACCAC
	Reverse	ACCTTAAGATTCAACCGCCAT
GAPDH (q-PCR)	Forward	CCTCTGACTTCAACAGCGACACC
	Reverse	ACCACCCTGTTGCTGTAGCCAA
PKM (RT-PCR)	Forward	CTGAAGGCAGTGATGTGGCC
	Reverse	ACCCGGAGGTCCACGTCCTC
SAM68 (gDNA-RT-PCR)	Forward	TGACTATTCTACAGTAGGCAT
	Reverse	CATTCTGCTCCTTAGGGTT
SAM68-Flag	Forward	ttaaacttaagcttggtaccATGCAGCGCCGGGACGAC

	Reverse	tcatcgtcgtccttgtagtcATAACGTCCATATGGGTGCTCTC
hnRNP A1-HA	Forward	ttaaacttaagcttggtaccATGTCTAAGTCAGAGTCTCCTAAAGAGC
	Reverse	tctggaacatcgtatgggtaAAATCTTCTGCCACTGCCATAGC
hnRNP A2/B1-HA	Forward	ttaaacttaagcttggtaccATGGAGAAAACTTTAGAAACTGTTCCT
	Reverse	tctggaacatcgtatgggtaGTATCGGCTCCTCCCACCA
hnRNP A1- MUT1- HA	Forward	ttaaacttaagcttggtaccATGTCTAAGTCAGAGTCTCCTAAAGAGC
	Reverse	tctggaacatcgtatgggtaTGGTTCCACAACTCTTCCATCC
hnRNP A1- MUT2- HA	Forward	ttaaacttaagcttggtaccATGTCTAAGTCAGAGTCTCCTAAAGAGC
	Reverse	tctggaacatcgtatgggtaTTGCTTTGACAGGGCTTTTCTAA
hnRNP A1- MUT3- HA	Forward	ttaaacttaagcttggtaccATGTCTAAGTCAGAGTCTCCTAAAGAGC
	Reverse	tctggaacatcgtatgggtaACTGCCACCATATCCACCACC
hnRNP A1- MUT4- HA	Forward	ttaaacttaagcttggtaccATGGAACCAAAGAGAGCTGTCTCC
	Reverse	tctggaacatcgtatgggtaAAATCTTCTGCCACTGCCATAGC
hnRNP A1^{RAAMUT}- HA	Forward-1	ggtgggaatgacaacttcggtcgtgcagcaAACTTCAGTGGTCGTGGTGGC
	Reverse-1	gaagttgtcattcccaccgaaacctgctgcACGACCACCACCAAAGTTTCC
	Forward-2	atttggtggcagccgtgcagcaGGTGGATATGGTGGCAGTGG

	Reverse-2	cacggctgccaccaaatgctgcACGACCACTGAAGTTTGCTGC
FN-1	Forward	GAAGAGCGAGCCCCTGATTGG
	Reverse	CACCTACATTCGGCGGGTATG
ZNF621	Forward	ACCAGCTCCTCGGCGTTCTG
	Reverse	TTGTTTGGAGCATGGCGGATG
ACADVL	Forward	TCCCATACCCGTCCGTGCTCA
	Reverse	CTCCACCATCTCCAGAGCGTCA
OSBPL1A-Exon 5-6	Forward	TCCGAGTCCGAAAGGT
	Reverse	GAGGATGCTCCAGATA
OSBPL1A-Exon 6-8	Forward	AAGTTTGCCTTCTCCTATGTT
	Reverse	TTTCCAGTCCGTTCCC
OSBPL1A-Exon 5-7	Forward	GAGAGCATTTGGGCAGTAGAA
	Reverse	TTAGGCGCTGTAGGAA
OSBPL1A-Exon 7-8	Forward	GAGCCTCTGAGCTTCCTACAG
	Reverse	GTTTTCCAGTCCGTTCC
ANAPC11	Forward	GCTGTTGAGGGAGTCGG
	Reverse	ATGCCACAGTTCTCATCGTTG
**Oligonucleotides**		
siSAM68#1:	sense:5‘-CCUGCACCAGAAACAUACGAAGAUU -3‘,	
siSAM68#2:	sense: 5‘- GAGAGCAUCCAUAUGGACGUUAUUA -3‘,	
sihnRNP A1#1:	sense: 5‘- GUGUAGUUGAACUGAUAGUTT -3‘,	
sihnRNP A1#2:	sense: 5‘- GCUGUGUAAAGUUAGUCUATT -3‘,	
siPKM2:	sense: 5‘- GCCAUCUACCACUUGCAAUTT -3‘,	
siNC:	sense: 5‘-GCACAAGCUGGAGUACAACUACATT-3‘	
EI9(50-68)	Biotin-AGGUAGGGCCCUAAGGGCA	
EI9(50-68, G3C)	Biotin-AGGUACGGCCCUAAGGGCA	

**SAM68 Cas9/sgRNAs**	
Site 1	5‘-TACGCAGAACAA AGTTACGA-3‘
Site 2	5‘-TGACTCTGGCTGTAATAGCC-3‘
